# Randomized Incremental Construction of Delaunay Triangulations of Nice Point Sets

**DOI:** 10.1007/s00454-020-00235-7

**Published:** 2020-09-08

**Authors:** Jean-Daniel Boissonnat, Olivier Devillers, Kunal Dutta, Marc Glisse

**Affiliations:** 1 Université Côte d’Azur, INRIA Sophia-Antipolis, Sophia-Antipolis, France; 2 INRIA, CNRS, Loria, Université de Lorraine, Nancy, France; 3 Department of Informatics, University of Warsaw, Warsaw, Poland; 4 INRIA, Université Paris-Saclay, Île-de-France, France

**Keywords:** Randomized incremental construction, Delaunay triangulations, Voronoi diagrams, Flat torus, Polyhedral surfaces, Probabilistic analysis, 52-08, 52C45, 52C15, 52C17, 68Q87

## Abstract

*Randomized incremental construction* (RIC) is one of the most important paradigms for building geometric data structures. Clarkson and Shor developed a general theory that led to numerous algorithms which are both simple and efficient in theory and in practice. Randomized incremental constructions are usually space-optimal and time-optimal in the worst case, as exemplified by the construction of convex hulls, Delaunay triangulations, and arrangements of line segments. However, the worst-case scenario occurs rarely in practice and we would like to understand how RIC behaves when the input is nice in the sense that the associated output is significantly smaller than in the worst case. For example, it is known that the Delaunay triangulation of nicely distributed points in ${\mathbb {E}}^d$ or on polyhedral surfaces in ${\mathbb {E}}^3$ has linear complexity, as opposed to a worst-case complexity of $\Theta (n^{\lfloor d/2\rfloor })$ in the first case and quadratic in the second. The standard analysis does not provide accurate bounds on the complexity of such cases and we aim at establishing such bounds in this paper. More precisely, we will show that, in the two cases above and variants of them, the complexity of the usual RIC is $O(n\log n)$, which is optimal. In other words, without any modification, RIC nicely adapts to good cases of practical value. At the heart of our proof is a bound on the complexity of the Delaunay triangulation of random subsets of ${\varepsilon }$-nets. Along the way, we prove a probabilistic lemma for sampling without replacement, which may be of independent interest.

## Introduction

The *randomized incremental construction* (RIC) is an algorithmic paradigm introduced by Clarkson and Shor [12], which has since found immense applicability in computational geometry, e.g. [27, 28]. The general idea is to process the input points sequentially in a random order, and to analyze the expected complexity of the resulting procedure. The theory developed by Clarkson and Shor is quite general and led to numerous algorithms which are simple and efficient, both in theory and in practice. On the theory side, randomized incremental constructions are usually optimal in space and time in the worst case, as exemplified by the construction of convex hulls, Delaunay triangulations, and arrangements of line segments. Randomized incremental constructions are also known to perform very efficiently in practice, which, together with their simplicity, makes them the most popular candidates for implementations. Not surprisingly, the cgal library includes several randomized incremental algorithms, e.g. for computing Delaunay triangulations.

Experimental evidence has shown that randomized incremental constructions often work much better than the worst-case analysis suggests, which is fortunate since worst-case situations are rare in applications. This paper aims at understanding how randomized incremental constructions behave, when the input is nice in the sense that the associated construction is significantly smaller than in the worst case.

We need a model of good point sets to describe the input data and analyze the algorithms. This will be done through the notion of ${\varepsilon }$-nets, which have a long and rich history since their introduction in the 1950s in the works of Kolmogorov and others on functional analysis and topological vector spaces (see e.g. [31]). ${\varepsilon }$-Nets have become ubiquitous in many theoretical as well as applied areas, from geometry and functional analysis to probability theory and statistics, where they are often used as countable or finite approximations of continuous spaces.

When we work with such a hypothesis of “nice” distribution of the points in space, a volume counting argument ensures that the local complexity of the Delaunay triangulation around a vertex is bounded by a constant (dependent only on the dimension). Unfortunately, to be able to control the complexity of the usual randomized incremental algorithms [3, 10, 12, 15], it is not enough to control the final complexity of the Delaunay triangulation. We need to control also the complexity of the triangulation of random subsets. One might expect that a random subsample of size *k* of an ${\varepsilon }$-net is also an ${\varepsilon }'$-net for ${\varepsilon }'={\varepsilon }\root d \of {{n}/{k}}$. Actually this is not quite true, it may happen with reasonable probability that a ball of radius $O({\varepsilon }')$ contains $\Omega (\log k / \log \log k)$ points or that a ball of radius $\Omega ({\varepsilon }'\root d \of {\log k})$ does not contain any point. For the convenience of the reader, we briefly sketch the proofs in the appendix (Lemma A.1). However, it can only be shown that such a subsample is an $({{\varepsilon }'}/({\log \,(1/{\varepsilon })})$-covering and an $({\varepsilon }'\log (1/{\varepsilon }))$-packing, with high probability. Thus this approach can transfer the complexity of an ${\varepsilon }$-net to the one of a random subsample of an ${\varepsilon }$-net but with an extra multiplicative factor of $\Omega (\log 1/{\varepsilon })=\Omega (\log n)$. It follows that, in the two cases we consider, the standard analysis does not provide accurate bounds on the complexity of the (standard) randomized incremental construction. Our results are based on proving that, *in expectation*, the above bad scenarios occur rarely, and the algorithm achieves optimal time complexity.

**Related work** The Delaunay triangulations of nicely-distributed points have been studied since the 50s, e.g. in the work of Meijering [22], Gilbert [20], Miles [23], and Møller [26]. More recently, Dwyer [16, 17] investigated Delaunay triangulations for uniformly distributed points, Golin and Na [21] studied the case of Poisson-distributed points. Moving on to deterministic point distributions, Attali and Boissonnat [4], Attali et al. [5], Amenta et al. [1], and others considered $({\varepsilon },\kappa )$-samples for various surfaces, and Erickson [18, 19] studied points with bounded spread (the ratio between the maximum to minimum distance between any two points). Except for a few authors such as Dwyer [16] and Erickson [19], most of the above results discuss only the combinatorial aspects and not the algorithmic ones. For Poisson and uniformly distributed point samples, we observe that the standard analysis of the RIC procedure immediately implies a bound on the expected running time, which is (up to a constant factor) the expected number of simplices times a logarithmic factor, and this is optimal. However, for deterministic notions of nice distributions such as ${\varepsilon }$-nets, $({\varepsilon },\kappa )$-samples, and bounded spread point sets, the standard RIC analysis is not optimal, since, as we observed, it gives at least an extra logarithmic factor for $({\varepsilon },\kappa )$-samples and even worse for bounded spread point sets, as stated in an open problem by Erickson [19]. Miller et al. [25] follow a very different approach, giving an algorithm to compute the approximate Delaunay graph of a nicely-spaced superset of points for an arbitrary input point set, with optimal time complexity and a $2^{O(d)}$ dependence on the dimension. Miller and Sheehy [24] give an algorithm for point sets with bounded spread, with a similar strategy which computes Voronoi diagrams with a run-time depending logarithmically on the spread. However, these algorithms are rather complicated and use several subroutines that have varying difficulties of implementation. Although ${\varepsilon }$-nets have a bounded spread, the RIC, while having a worse $2^{O(d^2)}$ dependence on the dimension, computes the entire Delaunay triangulation of the given point set rather than a superset, is easy to implement, and works efficiently in practice. As Miller et al. observe in [25], the quadratic dependence on the dimension in the exponent may be impossible to avoid for computing the exact Delaunay graph.

**Our contribution** We consider two main questions in this paper. First, we consider the case of an ${\varepsilon }$-net in the periodic space of dimension *d*, which, as mentioned before, has linear complexity instead of the worst-case $\Theta (n^{\lfloor d/2\rfloor })$. The reason to consider a periodic space is to avoid dealing with boundary effects that would distract us from the main point, and the fact that periodic spaces are often used in practice, e.g. in simulations in astronomy, biomedical computing, solid-state chemistry, condensed matter physics, etc. [11, 13, 20, 29, 33]. Following this, we deal with ${\varepsilon }$-nets on a polyhedral surface of ${\mathbb {E}}^3$, which is also a commonly-occurring practical scenario in e.g. surface reconstruction [2, 8], and has Delaunay triangulations with linear complexity, as opposed to quadratic bounds in the worst-case scenario. In this case, the boundary effects need to be explicitly controlled, which requires a more careful handling along with some new ideas. In both cases, we establish tight bounds and show that the complexity of the usual RIC is $O(n\log n)$, which is optimal. Hence, without any modification, the standard RIC nicely adapts to the good cases above. Our technical developments rely on a general bound for the probability of certain non-monotone events in sampling without replacement, which may be of independent interest.

**Extensions** We also give some extensions of our results for periodic spaces. Our extensions are in four directions: (i) a more general notion of well-distributed point sets, the $({\varepsilon },\kappa )$-samples; (ii) a different notion of subsampling—the Bernoulli or i.i.d. sample where each point is selected to be in ${{\mathcal {Y}}}$ independently of the others, with probability $q=s/n$, (iii) a more general class of spaces—Euclidean *d*-orbifolds, and (iv) a more general class of metrics—those having bounded-distortion with respect to the Euclidean metric. Precisely, for all the above cases, we show that the Delaunay triangulation of a random subsample has a linear size in expectation. We believe that our methods should work for an even larger class of spaces, though this might require more delicate handling of boundary effects and other features specific to the metric space under consideration.

**Outline** The rest of the paper is as follows. In Sect. 2, we define the basic concepts of Delaunay triangulation, ${\varepsilon }$-net, flat torus, and random samples. We state our results in Sect. 3. In Sect. 4, we bound the size of the Delaunay triangulation of a uniform random sample of a given size extracted from an ${\varepsilon }$-net on the flat torus ${\mathbb {T}}^d$. In Sect. 5, we analyze the case when the uniform random subsample is drawn from an ${\varepsilon }$-net on a polyhedral surface in ${\mathbb {E}}^3$. In Sect. 6, we use the size bounds established in Sects. 4 and 5, to compute the space and time complexity of the randomized incremental construction for constructing Delaunay triangulations of ${\varepsilon }$-nets. Finally, in Sect. 7, we state and prove some extensions. Proofs missing in the main sections are given in the appendix.

## Background

### Notations

We denote by $\Sigma (p,r)$, *B*(*p*, *r*) and *B*[*p*, *r*], the sphere, the open ball, and the closed ball of center *p* and radius *r* respectively. For $x\in {\mathbb {E}}^2$, $y\ge 0$, *D*(*x*, *r*) denotes the disk with center *x* and radius *r*, i.e., the set of points $\{y\in {\mathbb {E}}^2:\Vert y-x\Vert < r\}$, and similarly *D*[*x*, *r*] denotes the corresponding closed disk. The volume of the unit Euclidean ball of dimension *d* is denoted $V_d$ and the area of the boundary of such a ball is denoted $S_{d-1}$. It is known that $V_d={\pi ^{d/2}}\,/\,{\Gamma \,({{d}/{2}+1})}$ and $S_d=2\pi \, V_{d-1}$, where $\Gamma (t)= \int _0^\infty e^{-x}x^{t-1}\,dx$, $t> 0$, denotes the *gamma function*. For $d \in {\mathbb {Z}}^+$, $\Gamma (d+1) = d!$  We note that $2^{d}d^{-d/2}\le V_d \le 2^{4d}d^{-d/2}$ (see e.g. [35]).

For an event ${\mathcal {E}}$ in some probability space $\Omega$, we use ${\mathbf {1}}_{[{\mathcal {E}}]}$ to denote the indicator variable ${\mathbf {1}}_{[{\mathcal {E}}]}={\mathbf {1}}_{[{\mathcal {E}}]}(\omega )$ which is 1 whenever $\omega \in {\mathcal {E}}$, and 0 otherwise. We use [*n*] to mean the set $\{1,2,\ldots ,n\}$. Given a discrete set *A*, $\sharp (A)$ denotes its cardinality and, for $k\in {\mathbb {Z}}^+$, ${A\atopwithdelims ()k}$ denotes the set of *k*-sized subsets of *A*. Given an event *A* in some probability space, $\mathbb {P}[A]$ denotes the probability of *A* occurring. For a random variable *Z* in a probability space, $\mathbb {E}[Z]$ denotes the expected value of *Z*. Lastly, $e=2.7182\ldots$ denotes the base of the natural logarithm.

### ${\varepsilon }$-Nets

A set ${{\mathcal {X}}}$ of *n* points in a metric space $\mathcal{M}$, is an ${\varepsilon }$*-packing* if any pair of points in ${{\mathcal {X}}}$ are at least distance ${\varepsilon }$ apart, and an ${\varepsilon }$-*cover* if each point in $\mathcal{M}$ is at distance at most ${\varepsilon }$ from some point of ${{\mathcal {X}}}$. The set ${{\mathcal {X}}}$ is an ${\varepsilon }$-*net* if it is an ${\varepsilon }$-cover and an ${\varepsilon }$-packing simultaneously. The definition of an ${\varepsilon }$-net applies to any metric space, but in the case of Euclidean metric, some additional properties can be proven. We shall use $\Vert \,{\cdot }\,\Vert$ to denote the Euclidean $\ell _2$ norm. The following lemmas are folklore.

#### Lemma 2.1

(Maximum packing size) Any packing of the ball of radius $r\ge \rho$ in dimension *d* by disjoint balls of radius $\rho /2$ has a number of balls smaller than $({3r}/{\rho })^d$.

#### Proof

Consider a maximal set of disjoint balls of radius ${\rho }/{2}$ with centers inside the ball *B*(*r*) of radius *r*. Then the balls with the same centers and radius $\rho$ cover the ball *B*(*r*) (otherwise it contradicts the maximality). By a volume argument we get that the number of balls is bounded from above by

$$\begin{aligned} \frac{V_d \cdot (r+{\rho }/{2})^d}{V_d\cdot ({\rho }/{2})^d}\le \biggl (\frac{3r}{\rho }\biggr )^d. \end{aligned}$$

$$\square $$

#### Lemma 2.2

(Minimum cover size) Any covering of a ball of radius *r* in dimension *d* by balls of radius $\rho$ has a number of balls greater than $({r}/{\rho })^{d}$.

#### Proof

The volume argument gives a lower bound of ${V_d\cdot r^d}/({V_d \cdot \rho ^d})=({r}/{\rho })^{d}$. $\square$

For $d\in {\mathbb {Z}}^+$, the *flat* *d*-*torus* ${\mathbb {T}}^d$ is the compact quotient group ${\mathbb {E}}^d/{\mathbb {Z}}^d$, with addition as the group action. More generally, for $k\in {\mathbb {Z}}^+$, the flat torus of length *k* is ${\mathbb {T}}_k^d:= {\mathbb {E}}^d/(k{\mathbb {Z}})^d$.

#### Lemma 2.3

$({\varepsilon }$-Net size bounds) Given ${\varepsilon }\in (0,1/2]$, let ${{\mathcal {X}}}$ be an ${\varepsilon }$-net over the flat torus ${\mathbb {T}}^d$. Then, $\sharp ({{\mathcal {X}}})\in \bigl [ d^{d/2}2^{-4d}{\varepsilon }^{-d},\,d^{d/2}{\varepsilon }^{-d}\bigr ]$.

#### Proof

Observe that, by the minimum distance property of the points in ${{\mathcal {X}}}$, the balls of radius ${\varepsilon }/2$ centered at each point in ${{\mathcal {X}}}$ are disjoint, and by a volume argument there can be at most

$$\begin{aligned} \frac{1}{V_d\cdot ({\varepsilon }/2)^d}\le \frac{d^{d/2}}{ 2^d\cdot ({\varepsilon }/2)^d}=\frac{d^{d/2}}{{\varepsilon }^d} \end{aligned}$$

such balls in ${\mathbb {T}}^d$. The balls of radius ${\varepsilon }$ centered at each point in ${{\mathcal {X}}}$ cover the space thus their number is at least

$$\begin{aligned} \frac{1}{V_d\cdot {\varepsilon }^d}\ge \frac{d^{d/2}}{2^{4d}\cdot {\varepsilon }^d}. \end{aligned}$$

This completes the proof of the lemma. $\square$

### Delaunay Triangulation

For simplicity of exposition and no real loss of generality, all finite point sets considered in this paper will be assumed to be in *general position*, i.e., no set of $d+2$ points lie on a sphere. Given a set ${{\mathcal {X}}}$ in some ambient topological space, the *Delaunay complex* of ${{\mathcal {X}}}$ is the (abstract) simplicial complex with vertex set ${{\mathcal {X}}}$ which is the nerve of the Voronoi diagram of ${{\mathcal {X}}}$, that is, a simplex $\sigma$ (of arbitrary dimension) belongs to ${\text {Del}}({{\mathcal {X}}})$ iff the Voronoi cells of its vertices have a nonempty common intersection. Equivalently, $\sigma$ can be circumscribed by an empty ball, i.e., a ball whose bounding sphere contains the vertices of $\sigma$ and whose interior contains no points of ${{\mathcal {X}}}$.

The Delaunay complex is a triangulation if it triangulates the ambient space, or more precisely, the Delaunay complex ${\text {Del}}({{\mathcal {X}}})$ of a point set ${{\mathcal {X}}}$ over an ambient space $\mathcal{M}$, is said to be a *Delaunay triangulation* of $\mathcal{M}$ if there exists a homeomorphism between ${\text {Del}}({{\mathcal {X}}})$ and $\mathcal{M}$. The *combinatorial complexity* of a Delaunay triangulation is the total number of simplices of all dimensions, contained in the triangulation. The combinatorial complexity of a Delaunay triangulation is at most $2^d$ times the number of maximum-dimensional simplices contained in it.

Given a set ${{\mathcal {X}}}$ in some ambient space $\mathcal{M}$, with its Delaunay complex ${\text {Del}}({{\mathcal {X}}})$, the *star* of a subset $S\in {{\mathcal {X}}}$, or ${\text {star}}(S)$, is the set of all simplices in ${\text {Del}}({{\mathcal {X}}})$ that are incident to at least one point in *S*. For a point $p\in {{\mathcal {X}}}$, we shall use the shorthand expression ${\text {star}}(p)$ to mean ${\text {star}}(\{p\})$.

Given topological spaces $\mathcal{S}$ and $\mathcal{C}$, and a continuous map $\pi :\mathcal{C}\rightarrow \mathcal{S}$, $\mathcal{C}$ is a *covering space* of $\mathcal{S}$ if $\pi$ is such that for every point $x \in \mathcal{S}$, there exists an open neighborhood *U* of *x*, such that the pre-image $\pi ^{-1}(U)$ is a disjoint union of open neighborhoods in $\mathcal{C}$, each of which is homeomorphically mapped onto *U* by $\pi$. A covering $\mathcal{C}$ of $\mathcal{S}$ is *m*-*fold* or *m*-*sheeted* if the cardinality of the pre-image of each point $x\in \mathcal{S}$ under the covering map is *m*. For example, ${\mathbb {T}}_k^d$ forms a $k^d$-sheeted *covering space* of ${\mathbb {T}}^d$, with the covering map $x\mapsto {x}{\mod 1}$, the modulus operation being defined coordinate-wise. Caroli and Teillaud [11] showed the following.

#### Theorem 2.4

The Delaunay complex of any finite point set in ${\mathbb {T}}^d$ having at least one point, embeds into the $3^d$-sheeted covering of ${\mathbb {T}}^d$. If the maximum circumradius of a simplex is at most 1/2, then the complex embeds into ${\mathbb {T}}^d$ itself.

Note that the above theorem implies that the Delaunay triangulation of any finite point set in ${\mathbb {T}}^d$ *always exists* in the $3^d$-sheeted covering of ${\mathbb {T}}^d$.

A key property of ${\varepsilon }$-nets is that their Delaunay triangulations have linear size.

#### Lemma 2.5

(Talmor [30]) Let ${\varepsilon }\in (0,1/2]$ be given, and let ${{\mathcal {X}}}$ be an ${\varepsilon }$-net over ${\mathbb {T}}^d$. Then the Delaunay triangulation of ${{\mathcal {X}}}$, ${\text {Del}}({{\mathcal {X}}})$, has complexity at most $4^{d^2}/{\varepsilon }^d$.

#### Proof

Let us first bound the number of *d*-dimensional simplices in ${\text {Del}}({{\mathcal {X}}})$. Observe that the circumradius of any *d*-simplex in ${\text {Del}}({{\mathcal {X}}})$ cannot be greater than ${\varepsilon }$, since this would imply the existence of a ball in ${\mathbb {T}}^d$ of radius at least ${\varepsilon }$, containing no points from ${{\mathcal {X}}}$. Therefore given a point $p\in {{\mathcal {X}}}$, any point that lies in a Delaunay simplex incident to *p*, must be at most of distance $2{\varepsilon }$ from *p*. Again by a volume argument, the number of such points is at most ${V_d\cdot (2{\varepsilon }+{\varepsilon }/2)^d}/({V_d\cdot ({\varepsilon }/2)^d}) = 5^d$. Thus, the number of Delaunay simplices of dimension *d* that contain *p*, is at most the complexity of the Delaunay triagulation in ${\mathbb {T}}^d$ on $5^d$ vertices. This is at most $(5^d)^{\lceil d/2\rceil }$. Thus we can conclude that the number of *d*-simplices in ${\text {Del}}({{\mathcal {X}}})$ is at most the cardinality of ${{\mathcal {X}}}$, times the maximum number of *d*-simplices incident to any given point $p\in {{\mathcal {X}}}$. Now using Lemma 2.3, together with the fact that the complexity is at most $2^d$ times the number of *d*-simplices, we get that

$$\begin{aligned} (5^d)^{\lceil d/2\rceil } \cdot 2^d\cdot \sharp ({{\mathcal {X}}})\le \frac{ (5^d)^{ ({d+1})/{2}}\cdot 2^d\cdot d^{d/2}}{ {\varepsilon }^d}\le \frac{4^{d^2} }{{\varepsilon }^d}. \end{aligned}$$

$$\square $$

### Randomized Incremental Construction and Random Subsamples

For the algorithmic complexity aspects, we state a version of a standard theorem for the RIC procedure (see e.g. [14]). We first need a necessary condition for the theorem. When a new point *p* is added to an existing triangulation, a *conflict* is defined to be a previously existing simplex whose circumball contains *p*.

#### Condition 2.6

At each step of the RIC, the set of simplices in conflict can be removed and the set of newly introduced conflicts computed in time proportional to the number of conflicts.

We now come to the general theorem on the algorithmic complexity of RIC using the Clarkson–Shor technique (see e.g. Devillers [14, Thm. 5 (1,2)]).

#### Theorem 2.7

Let *F*(*s*) denote the expected number of simplices that appear in the Delaunay triangulation of a uniform random sample of size *s*, from a given point set *P*. Then, if Condition 2.6 holds and $F(s)=O(s)$, we have:

(i) The expected space complexity of computing the Delaunay triangulation is *O*(*n*).
(ii) The expected time complexity of computing the Delaunay triangulation is $\sum _{s=1}^n ({n-s})/{s} = O(n\log n)$.

A subset ${{\mathcal {Y}}}$ of set ${{\mathcal {X}}}$ is a *uniform random sample* of ${{\mathcal {X}}}$ *of size s* if ${{\mathcal {Y}}}$ is any possible subset of ${{\mathcal {X}}}$ of size *s* with equal probabilities. In case the multiplicity of a point in ${{\mathcal {X}}}$ is greater than 1, the sample counts only one copy of the point; all other copies are present in ${{\mathcal {Y}}}$ if and only if the original point is present.

In order to work with uniform random samples, we shall prove a lemma on the uniformly random sampling distribution or *sampling without replacement*, which is stated below, and will be a key probabilistic component of our proofs. The lemma provides a bound on the probability of a non-monotone compound event, that is, if the event holds true for a fixed set of *k* points, there could exist supersets as well as subsets of the chosen set for which the event does not hold. This may well be of general interest, as most natural contiguity results with Bernoulli (i.e., independent) sampling are for monotone events.

#### Lemma 2.8

Let $a,b,c\in {\mathbb {Z}}^+$ with $2b\le a \le c$ and $t\le c$. Let *C* be a set, and *B* and *T* two disjoint subsets of *C*. If *A* is a random subset of *C*, chosen uniformly from all subsets of *C* having size *a*, the probability that *A* contains *B* and is disjoint from *T*, is at most

$$\begin{aligned} \biggl (\frac{a}{c}\biggr )^{b}\biggl (1-\frac{t}{c-b}\biggr )^{a-b} \le \, \biggl (\frac{a}{c}\biggr )^{b}\exp \biggl (-\frac{at}{2c}\biggr ), \end{aligned}$$

where *a*, *b*, *c* are the cardinalities of *A*, *B*, and *C* respectively, and the cardinality of *T* is at least *t*.

#### Proof

The total number of ways of choosing the random sample *A* is ${c\atopwithdelims ()a}$. The number of ways of choosing *A* so that $B\subset A$ and $T\cap A=\emptyset$, is ${c-b-t\atopwithdelims ()a-b}$. Therefore the required probability is

$$\begin{aligned}&{\mathbb {P}}\,[B\subset A, \, T\cap A = \emptyset ]\\&\quad =\frac{\displaystyle {c-b-t\atopwithdelims ()a-b}}{\displaystyle {c\atopwithdelims ()a}} = \frac{\displaystyle \prod _{i=0}^{b-1}(a-i) \prod _{i=b}^{a-1}(a-i)}{\displaystyle \prod _{i=0}^{b-1}(c-i) \prod _{i=b}^{a-1}(c-i)}\cdot \frac{\displaystyle \prod _{i=0}^{a-b-1}(c-b-t-i)}{\displaystyle \prod _{i=0}^{a-b-1}(a-b-i)} \\&\quad =\frac{\displaystyle \prod _{i=0}^{b-1}(a-i)}{\displaystyle \prod _{i=0}^{b-1}(c-i)}\,=\,\frac{\displaystyle \prod _{i=0}^{a-b-1}(c-b-t-i)}{\displaystyle \prod _{i=0}^{a-b-1}(c-b-i)} \\&\quad =\biggl (\frac{a}{c}\biggr )^{b}\cdot \,\frac{\displaystyle \prod _{i=0}^{b-1}\biggl (1-\frac{i}{a}\biggr )}{\displaystyle \prod _{i=0}^{b-1}\biggl (1-\frac{i}{c}\biggr )}\cdot \biggl (1-\frac{t}{c-b}\biggr )^{a-b}\cdot \,\frac{\displaystyle \prod _{i=0}^{a-b-1}\biggl (1-\frac{i}{c-b-t}\biggr )}{\displaystyle \prod _{i=0}^{a-b-1}\biggl (1-\frac{i}{c-b}\biggr )} \\&\quad \le \biggl (\frac{a}{c}\biggr )^{b}\biggl (1-\frac{t}{c-b}\biggr )^{a-b}, \end{aligned}$$

where in the last step observe that for the product

$$\begin{aligned} \frac{\displaystyle \prod _{i=0}^{b-1}\biggl (1-\frac{i}{a}\biggr )}{\displaystyle \prod _{i=0}^{b-1}\biggl (1-\frac{i}{c}\biggr )}, \end{aligned}$$

for each *i* the term $(1-i/a)$ in the numerator is smaller than the corresponding term $(1-i/c)$ in the denominator, since $a\le c$. A similar observation holds for the product

$$\begin{aligned} \frac{\displaystyle \prod _{i=0}^{a-b-1}\biggl (1-\frac{i}{c-b-t}\biggr )}{\displaystyle \prod _{i=0}^{a-b-1}\biggl (1-\frac{i}{c-b}\biggr )}. \end{aligned}$$

Now, observe that

$$\begin{aligned} \biggl (1-\frac{t}{c-b}\biggr )^{a-b}\le \, \exp \,\biggl ({-}\frac{t(a-b)}{c-b}\biggr ) \le \exp \,\biggl ({-}\frac{at}{2c}\biggr ), \end{aligned}$$

if $$b\le a/2$$ and $$b<c$$. $$\square $$

## Results

**Random samples of** ${\varepsilon }$-**nets in** ${\mathbb {T}}^d$ The following theorem gives a constant bound on the expected complexity of ${\text {star}}(p)$ for the Euclidean metric on the flat torus ${\mathbb {T}}^d$.

### Theorem 3.1

(Euclidean metric) Given an ${\varepsilon }$-net ${{\mathcal {X}}}$ in ${\mathbb {T}}^d$ in general position, where ${\varepsilon }\in (0,{1}/{4}]$, and a point $p\in {{\mathcal {X}}}$, the expected complexity of ${\text {star}}(p)$, ${{\,\mathrm{{\mathbb {E}}}\,}}\left[ \sharp ({\text {star}}(p))\right]$, in the Delaunay triangulation of a uniform random sample ${{\mathcal {Y}}}\subset {{\mathcal {X}}}$ of size $s\ge 4(2\sqrt{d})^{d}d^3+1$ containing *p*, is less than $2\cdot 6^{d^2+3d/2}$.

**Polyhedral surfaces in** ${\mathbb {E}}^3$ A *polyhedral surface* $\mathcal{S}$ in ${\mathbb {E}}^3$ is a collection of a finite number of polygons $F\subset \mathcal{S}$, called *facets*, which are pairwise disjoint or meet at a vertex or along an edge. We show that the expected complexity of the Delaunay triangulation of a uniformly random subsample of an ${\varepsilon }$-net on a polyhedral surface is linear in the size of the subsample:

### Theorem 3.2

Let ${\varepsilon }\in [0,1]$, ${{\mathcal {X}}}$ be an ${\varepsilon }$-net on a fixed polyhedral surface $\mathcal{S}$, with *C* facets having total area *A* and total length *L* along its boundaries, with *n* points and let ${{\mathcal {Y}}}\subset {{\mathcal {X}}}$ be a random sub-sample of ${{\mathcal {X}}}$ having size *s*. Then the Delaunay triangulation ${\text {Del}}({{\mathcal {Y}}})$ of ${{\mathcal {Y}}}$ on $\mathcal{S}$ has *O*(*s*) simplices.

**Algorithmic bounds** We next use the above combinatorial bounds to get the space and time complexity of the randomized incremental construction of the Delaunay triangulation of an ${\varepsilon }$-net on the flat *d*-torus or on a polyhedral surface in ${\mathbb {E}}^3$.

### Theorem 3.3

(Randomized incremental construction) Let ${\varepsilon }\in [0,1/4]$, and let ${{\mathcal {X}}}$ be an ${\varepsilon }$-net in general position over (i) the flat *d*-dimensional torus ${\mathbb {T}}^d$, or (ii) a fixed polyhedral surface $\mathcal{S}\subset {\mathbb {E}}^3$. Then the randomized incremental construction of the Delaunay triangulation takes $O(n\log n)$ expected time and *O*(*n*) expected space, where $n=\sharp ({{\mathcal {X}}})$ and the constant in the big *O* depends only on *d*, and not on *n* nor ${\varepsilon }$. Further, at each step of the randomized incremental construction, the Delaunay complex of the set ${{\mathcal {Y}}}$ of already added points of ${{\mathcal {X}}}$ is a triangulation of the space.

**Extensions** Finally, our extensions are stated and proven in Sect. 7.

## Euclidean Metric on ${\mathbb {T}}^d$

In this section, we prove that a subsample ${{\mathcal {Y}}}$ of a given size *s*, drawn randomly from an ${\varepsilon }$-net ${{\mathcal {X}}}\subset {\mathbb {T}}^d$, has a Delaunay triangulation in which the star of any given vertex has a constant expected complexity. Hence, the expected complexity of the triangulation is linear in the size of the subsample. The constant of proportionality is bounded by $2^{cd^2}$, where *c* is a constant independent of ${\varepsilon }$ and *d*.

**Existence of Delaunay triangulation** ${\text {Del}}({{\mathcal {Y}}})$: In order to ensure we always have the Delaunay complex embedded in ${\mathbb {T}}^d$, we shall use Theorem 2.4. Accordingly, we get two different regimes of candidate simplices in the triangulation. When the circumradius of a candidate simplex $\sigma \in {\text {star}}(p)$ is at most 1/4, then the simplex lies in a ball of radius at most 1/2 with center *p*. By Theorem 2.4, in this regime the Delaunay complex ${\text {star}}(p)$ embeds in the one-sheeted covering of ${\mathbb {T}}^d$. Therefore, for a fixed set of vertices, there is a unique circumball. When the circumradius is greater than 1/4, the simplex is contained in a ball of radius greater than 1/2 around *p*, and therefore ${\text {star}}(p)$ embeds in the $3^d$-sheeted covering of ${\mathbb {T}}^d$, i.e., ${\mathbb {T}}_3^d$. In this case, each vertex has $3^d$ copies, and so for a given choice of *d* vertices together with *p*, one can have $(3^{d})^d$ circumballs.

**Proof framework** Now we set up the formal proof. We first focus on bounding the expected number of *d*-simplices in ${\text {star}}(p)$. Recall that $n:= \sharp ({{\mathcal {X}}})$. Define $q:=({s-1})/({n-1})$ and $\delta :={\varepsilon }\cdot ({2d}/{q})^{1/d}$. Let $I_0:=[0,\delta )$, $I_k:= [2^{k-1}\delta ,2^k\delta )$ for $k> 0$. To bound the expected number of *d*-simplices, we shall consider the probability of presence in ${\text {Del}}({{\mathcal {Y}}})$, of *d*-simplices in ${{\mathcal {X}}}$ incident to *p* and having radius in the interval $I_k$, as *k* ranges over ${\mathbb {Z}}^+$.

Throughout this section, we shall use $\sigma$ to mean a *d*-simplex incident to *p*, with circumcenter $c_\sigma$ and circumradius $r_\sigma$. To count the number of *d*-simplices in ${\text {star}}(p)$ with circumradius in $I_k$, let $S_p(k)$ denote the set of *d*-simplices having *p* as a vertex, with circumradius $r_\sigma \in I_k$. Set $s_p(k):= \sharp (S_p(k))$. For $\sigma \in S_p(k)$, let $P_p(k)$ denote the upper bound on the probability that $\sigma$ appears in ${\text {Del}}({{\mathcal {Y}}})$, that is,

$$\begin{aligned} P_p(k) := \max _{\sigma \in S_p(k)}{\mathbb {P}}\,[\sigma \in {\text {Del}}({{\mathcal {Y}}})]. \end{aligned}$$

Finally, let $Z_p(k)$ denote the number of *d*-simplices $\sigma \in S_p(k)$ such that $\sigma \in {\text {Del}}({{\mathcal {Y}}})$. The main lemma in the proof is a bound on the expected complexity of the star of *p*, in terms of $s_p(k)$ and $P_p(k)$.

### Lemma 4.1

$${\mathbb {E}}\,[\sharp ({\text {star}}(p))] =\displaystyle \sum _{k\ge 0}{\mathbb {E}}\,[Z_p(k)]\le \sum _{k\ge 0} s_p(k)\,P_p(k)$$.

### Proof

For a simplex $\sigma \in S_p(k)$, let ${\mathbf {1}}_{[\sigma ]}$ be the indicator random variable which is 1 if $\sigma \in {\text {star}}(p)$, and zero otherwise. Then $Z_p(k) := \sum _{\sigma \in S_p(k)} {\mathbf {1}}_{[\sigma ]}$, and $\sharp ({\text {star}}(p)) = \sum _{k\ge 0} Z_p(k)$. Taking expectations over the random choice of ${{\mathcal {Y}}}$, we get

$$\begin{aligned} {\mathbb {E}}\,[\sharp (v(p))]&=\sum _{k\ge 0}{\mathbb {E}}[Z_p(k)]=\sum _{k\ge 0}\,\sum _{\sigma \in S_p(k)}{\mathbb {E}}[{\mathbf {1}}_{[\sigma ]}]\\&\le \sum _{k\ge 0}\,\sum _{\sigma \in S_p(k)} P_d(k)=\sum _{k\ge 0} s_p(k)\,P_d(k). \end{aligned}$$

$$\square $$

It only remains therefore, to establish bounds on $s_p(k)$ and $P_p(k)$ as functions of *k*, and finally to bound the sum $\sum _{k\ge 0}s_p(k)\,P_p(k)$. Following the earlier discussion on the existence of the Delaunay triangulation ${\text {Del}}({{\mathcal {Y}}})$, we shall split the sum $\sum _{k\ge 0} {\mathbb {E}}[Z_p(k)]$ into the two regimes, $0\le k\le k_{\max }$ and $k>k_{\max }$, where $k_{\max }$ denotes $\log _2 ({1}/{4\delta })$.

**Case I:** *d*-**simplices with small circumradii** $k\in [0,k_{\max }]$. In this regime, the circumradii $r_\sigma$ of the candidate *d*-simplices are at most 1/4 since, by the definition of $k_{\max }$, we have $r_\sigma \le 2^{k_{\max }}=1/4$. Therefore every set of $d+1$ vertices in ${{\mathcal {Y}}}$, has a unique circumball. We begin by establishing a bound on $P_p(k)$. Let $n_k$ denote the minimum number of points of ${{\mathcal {X}}}$ in the interior of a circumball of a *d*-simplex $\sigma \in S_p(k)$, over all $\sigma \in S_p(k)$: $n_k := \min _{\sigma \in S_p(k)}\sharp (B(c_\sigma ,r_\sigma )\cap {{\mathcal {X}}})$. First, we bound $n_k$ from below using Lemma 2.2.

### Lemma 4.2

Let $\sigma$ be a simplex incident to *p*, having circumradius $r_\sigma \in I_k$, $k\ge 0$. Then

$$\begin{aligned} n_k \ge {\left\{ \begin{array}{ll} 0, &{} k = 0,\\ ({{2^{k-1}\delta }/{{\varepsilon }}})^d, &{} 0 < k\le k_{\max }. \end{array}\right. } \end{aligned}$$

### Proof

When $k\le k_{\max }$, the radius of the circumball of a simplex $\sigma$ is at most $2^{k_{\max }}\delta \le 1/4<1/2$. Applying Theorem 2.4, we work in the one-sheeted covering of ${\mathbb {T}}^d$. Using the fact that ${{\mathcal {X}}}$ is an ${\varepsilon }$-covering, we apply Lemma 2.2 to get that $n_k \ge (2^{k-1}\delta /{\varepsilon })^d$. $\square$

Now applying Lemma 2.8, we can bound $P_p(k)$.

### Lemma 4.3

For $$k\ge 0$$, $$P_p(k) \le q^d\exp {({-}qn_k/2)}$$.

### Proof

The simplex $\sigma$ can be a Delaunay simplex only if (i) the set of its vertices is included in the subsample ${{\mathcal {Y}}}$, and (ii) all points in $B(c_\sigma ,r_\sigma )\cap {{\mathcal {X}}}$ are excluded from ${{\mathcal {Y}}}$. The idea is therefore, to use Lemma 4.2 to bound the number of points in $B(c_\sigma ,r_\sigma )\cap {{\mathcal {X}}}$ from below by $n_k$, and then upper bound the probability that all these points are excluded from ${{\mathcal {Y}}}$.

This suggests applying Lemma 2.8, with the universe having $c = n-1$ elements, sample size $a = s-1$, included set having $b=d$ elements, and excluded set having $t= n_k$ elements. We verify first that the conditions of the lemma are satisfied, i.e., (i) $b\le \min {\{{a}/{2},c-1\}}$, since $s \ge 4d + 1$. Now applying the lemma, we get

$$\begin{aligned} {\mathbb {P}}\,[\sigma \in {\text {Del}}({{\mathcal {Y}}})]&\le \biggl (\frac{s-1}{n-1}\bigg )^{d} \exp {\biggl (\frac{-n_k}{2}\cdot \frac{s-1}{n-1}\biggr )}\\&= q^d\exp \frac{-qn_k}{2}\le q^d\exp \frac{-qn_k}{2}, \end{aligned}$$

where the equality was by the substitution $q=({s-1})/({n-1})$, and the last inequality followed from the fact that $d = b \le c-1 = n-2$. $\square$

Next, we shall upper bound $s_p(k)$ from above. We first state a simple observation.

### Lemma 4.4

Let $\sigma$ be a *d*-simplex incident to *p*, with circumcenter $c_\sigma$ and circumradius $r_\sigma$. Then $\sigma \subset B[p,2r_\sigma ]$ and $B(c_\sigma ,r_\sigma ) \subset B(p,2r_\sigma )$.

### Proof

This follows simply from the triangle inequality. For the first statement, we have that for any $p'\in \sigma$, $\Vert p,p'\Vert \le \Vert p,c_\sigma \Vert + \Vert c_\sigma , p'\Vert = 2r_\sigma$. The second statement follows by replacing the above inequalities with strict inequalities for the points in the open ball $B(c_\sigma ,r_\sigma )$. $\square$

Now we can bound $s_p(k)$ using the above observation together with Lemma 2.1.

### Lemma 4.5

For $$k\ge 0$$, $$s_p(k) \le {(6\cdot 2^{k}\delta /{\varepsilon })^{d^2}}/{d!}$$

### Proof

Let $\sigma$ be an element of $S_p(k)$. Using Lemma 4.4 and the definition of $S_p(k)$, we have that $\sigma \subset B[p,2^{k+1}\delta ]$. If $k\le k_{\max }$, then $2^{k+1}\delta \le 1/2$. Therefore applying Theorem 2.4, we can work in the one-sheeted covering of ${\mathbb {T}}^d$. Now applying Lemma 2.1, the number of points in $B(p,2^{k+1}\delta )\cap {{\mathcal {X}}}$ is at most $(3\cdot 2^{k+1}\delta /{\varepsilon })^d = (6\cdot 2^k\delta /{\varepsilon })^d$. Therefore, the set of possible *d*-simplices incident to *p* and having vertices in $B[p,2^{k+1}\delta ]\cap {{\mathcal {X}}}$ is at most the set of all *d*-tuples of points in $B[p,2^{k+1}\delta ]\cap {{\mathcal {X}}}$, i.e., at most $(6\cdot 2^k\delta /{\varepsilon })^{d^2}/d!$ $\square$

Next, using the above bounds on values $s_p(k)$ and $n_k$, we shall bound on the sum $\sum _{k=0}^{k_{\max }}{\mathbb {E}}[Z_p(k)]$ in the following three lemmas.

### Lemma 4.6

$${\mathbb {E}}[Z_p(0)]\le 6^{d^2+d}$$.

### Proof

Substituting the bounds on $s_p(0)$ and $P_p(0)$ proven in Lemmas 4.2, 4.3, and 4.5, we have

$$\begin{aligned} {\mathbb {E}}[Z_p(0)]=s_p(0)\,P_p(0)\le \frac{(6\,(\delta /{\varepsilon }))^{d^2}}{d!}\cdot q^d\le \frac{6^{d^2}\cdot (2d)^{d}}{d!}\le 6^{d^2}\cdot (2e)^d, \end{aligned}$$

where in the second inequality we used the definition of $\delta$ to get $q(\delta /{\varepsilon })^d=2d$, and in the last inequality we used Stirling’s approximation $d^d/d! \le e^d$, and the fact that $2e<6$. $\square$

### Lemma 4.7

$$\begin{aligned} \sum _{k=0}^{k_{\max }}{\mathbb {E}}[Z_p(k)]\le \frac{6^{d^2+d}}{1-(2/e)^6}. \end{aligned}$$

### Proof

First, recall from Lemma 4.1 that $\sum _{k\ge 1}{\mathbb {E}}[Z_p(k)]\le \sum _{k\ge 0} s_p(k)\, P_p(k)$. Now from Lemmas 4.2, 4.3, and 4.5, we have that for all $k\ge 0$,

- $$s_p(k)\le \tilde{s}_p(k):= (6\cdot 2^k\delta /{\varepsilon })^{d^2}/d!$$ and
- $$P_p(k)\le \tilde{P}_p(k) := {\left\{ \begin{array}{ll} q^d,&{}k=0,\\ q^d\exp {({-}q(2^{k-1}\delta /{\varepsilon })^d/2)}, &{} k\in [1,k_{\max }]. \end{array}\right. }$$

Observe that $\sum _{k\ge 0} s_p(k)\,P_p(k) \le \sum _{k\ge 0} \tilde{s}_p(k)\, \tilde{P}_p(k)$. In order to bound $\sum _{k\ge 0} s_p(k) P_p(k)$, it therefore suffices to simply bound $\sum _{k\ge 0} \tilde{s}_p(k)\, \tilde{P}_p(k)$. For the rest of the proof, therefore, we shall focus on bounding this sum.

From the analysis, it will be easy to see that the close neighbor vertices will form the bulk of the *d*-simplices in ${\mathbb {E}}\,[{\text {star}}(p)]$, and the contribution of vertices farther from *p* will decrease exponentially with the distance.

**When** $1\le k\le k_{\max }$ Consider the ratio of successive terms of $(\tilde{s}_p(k)\, \tilde{P}_p(k))_{k\ge 1}$. From Lemmas 4.2 and 4.5, we have

$$\begin{aligned}&\frac{\tilde{s}_p(k+1)\, \tilde{P}_p(k+1)}{\tilde{s}_p(k)\, \tilde{P}_p(k)} = \frac{(6\cdot 2^{k+1}\delta /{\varepsilon })^{d^2}\,/d!}{(6\cdot 2^k\delta /{\varepsilon })^{d^2}\,/d!} \cdot \frac{q^d\exp {({-}q(2^{k}\delta /{\varepsilon })^d/2)}}{q^d\exp {({-}q(2^{k-1}\delta /{\varepsilon })^d/2)}}\\&\qquad = 2^{d^2}\exp \frac{-q(2^{k-1}\delta /{\varepsilon })^d(2^d-1)}{2}= 2^{d^2}\exp \frac{-2^{(k-1)d}2d(2^d-1)}{2}\le 2^4e^{-6}, \end{aligned}$$

where we used the definition of $\delta = {\varepsilon }(2d/q)^{1/d}$, i.e., $q(\delta /{\varepsilon })^d=2d$. The last step follows by taking $k=1$, $d=2$, to get $2^4\cdot e^{-6} \le (2/e)^6$.

**When** $k=0$ In this case, the ratio

$$\begin{aligned} \frac{\tilde{s}_p(1)\, \tilde{P}_p(1)}{\tilde{s}_p(0)\, \tilde{P}_p(0)} \le 2^{d^2}\exp {({-}d\cdot 2^d)}, \end{aligned}$$

which is at most $(2/e)^6$ for $d\ge 2$. Therefore for all $0\le k\le k_{\max }-1$, we have that

$$\begin{aligned} \frac{\tilde{s}_p(k+1)\, \tilde{P}_p(k+1)}{\tilde{s}_p(k)\, \tilde{P}_p(k)} \le \biggl (\frac{2}{e}\biggr )^{6}. \end{aligned}$$

Thus, the sum $\sum _{k=0}^{k_{\max }} {\mathbb {E}}[Z_p(k)]$ is upper bounded by the sum of a geometric progression with leading term $\tilde{s}_p(0)\, \tilde{P}_p(0)\le 6^{d^2+d}$ and common ratio $(2/e)^6$, which is at most $(1-(2/e)^6)^{-1}\cdot 6^{d^2+d}$. In other words, most of the Delaunay neighbors of *p* are the points nearest to *p*. $\square$

Lastly, we shall show that the expected number of *d*-simplices with exceptionally large circumradii, i.e., $\sum _{k>k_{\max }}{\mathbb {E}}[Z_p(k)]$, is at most a small constant.

**Case II:** *d*-**simplices with large circumradii** $k>k_{\max }$. In this regime, the circumradii of the candidate *d*-simplices are greater than 1/4. Therefore, by Theorem 2.4, we shall work in the $3^d$-sheeted covering of ${\mathbb {T}}^d$.

### Lemma 4.8

$$\begin{aligned} \sum _{k>k_{\max }}{\mathbb {E}}[Z_p(k)]\le 5. \end{aligned}$$

### Proof

From Lemma 2.3, we have that $n=\sharp ({{\mathcal {X}}})\le 2^{-d}d^{d/2}{\varepsilon }^{-d}$. Therefore, by Lemma 2.2, any ball *B* of radius at least $2^{k_{\max }}\delta \ge 1/4$, has at least $(2^{k_{\max }}\delta /{\varepsilon })^d =1/(4{\varepsilon })^d \ge {n}/({2^dd^{d/2}})$ points in its interior, i.e., $\sharp ({{\mathcal {X}}}\cap {{\,\mathrm{\mathrm{int}}\,}}B)\ge {n}/{(2\sqrt{d})^{d}}$. Here, since $2^{k+1}\delta > 1/2$, we shall use Theorem 2.4 and work in the $3^d$-sheeted covering of ${\mathbb {T}}^d$. The maximum number of *d*-tuples which can possibly form a Delaunay *d*-simplex with *p*, is at most ${n-1\atopwithdelims ()d} \le (n-1)^d/d!$ Since we are working in the $3^d$-sheeted covering space, each vertex of a simplex $\sigma \in S_p(k)$ can be chosen from one of at most $3^d$ copies in the covering space. Thus, each simplex in $S_p(k)$ yields less than $3^{d^2}$ possible Delaunay spheres in ${\mathbb {T}}^d$. Therefore, the expected number of *d*-simplices having radius at least $2^{k_{\max }}\delta$, is at most

1 $$\begin{aligned} \begin{aligned} \sum _{k>k_{\max }}{\mathbb {E}}[Z_p(k)]&=3^{d^2} \frac{(n-1)^d}{d!}\cdot P_p(k)=3^{d^2} \frac{(n-1)^d}{d!}\,q^d\exp {\biggl (-\frac{q}{2}\cdot \frac{n-1}{(2\sqrt{d})^d}\biggr )}\\&\le 3^{d^2} \frac{(s-1)^d}{d!}\exp {\biggl (-\frac{s-1}{2(2\sqrt{d})^d}\biggr )}. \end{aligned} \end{aligned}$$

For $s>s_0 =4(2\sqrt{d})^d\cdot d^3+1$, the bound (1) is a decreasing function of *s*, and it is easy to check that the value at $s_0$ is smaller than 5. The lemma follows. $\square$

Thus, by Lemmas 4.1, 4.7, and 4.8, the expected number of *d*-simplices in the star of *p* is at most $(1-(2/e)^6)^{-1}\cdot 6^{d^2+d}+5\le 2\cdot 6^{d^2+d}$ for $d\ge 2$. The expected complexity, therefore, is at most a factor $2^d$ times this, i.e., $2\cdot 6^{d^2+d}\cdot 2^d \le 2\cdot 6^{d^2+3d/2}$. This completes the proof of Theorem 3.1.

## Polyhedral Surfaces in ${\mathbb {E}}^3$

In this section, we introduce a partition of the sub-sample ${{\mathcal {Y}}}$ into *boundary* and *interior* points, to do a case analysis of the expected number of edges in the Delaunay triangulation ${\text {Del}}({{\mathcal {Y}}})$, depending on whether the end-points of a potential Delaunay edge are boundary or interior points, and whether they lie on the same facet or on different facets.

**Main ideas** Our overall strategy will be to mesh the proofs of Attali–Boissonnat [4] and Theorem 3.1. Briefly, Attali and Boissonnat reduce the problem to counting the Delaunay edges of the point sample, which, up to a constant factor, is equal to the number of simplices (see the proof of Theorem 3.2). They then count the edges by distinguishing between *boundary* and *interior* points of a facet. For boundary points, they allow all possible edges. For interior points, the case of edges with end-points on the same facet is easy to handle, while geometric constructions are required to handle the case of end-points on different facets, or that of edges with one end-point in the interior and another on the boundary.

However, we shall need to introduce some new ideas to adapt our previous methods to this setting. Firstly, for any pair of vertices, there are infinitely many balls containing the pair on the boundary, and as soon as one of these balls is empty (with respect to the points in ${{\mathcal {Y}}}$), the pair appears as an edge in the Delaunay triangulation. This is handled using a geometric construction (see Lemma 5.9). Basically, the idea is to build a constant-sized packing of a sphere centered on a given point, using large balls, such that any sphere of a sufficiently large radius which passes through the point, must contain a ball from the packing.

Secondly, since we no longer have a regular point distribution on the boundaries of $\mathcal{S}$, boundary effects could penetrate deep into the interior. To handle this, we introduce the notion of *levels* of a surface, instead of the fixed strip around the boundary used in [4], and use a probabilistic, rather than deterministic, classification of boundary and interior points. The new classification is based on the level of a point and the radius of the largest empty disk passing through it.

Recall the definitions of ${{\mathcal {X}}}$, ${{\mathcal {Y}}}$, and $\mathcal{S}$ from Theorem 3.2. We shall use $\kappa$ to denote the maximum number of points of a given point set in a disk of radius $2{\varepsilon }$. When ${{\mathcal {X}}}$ is an ${\varepsilon }$-net, $\kappa$ is at most $6^{d} =36$ (using Lemma 2.1 with $r=2{\varepsilon }$, $d=2$, and $\rho = {\varepsilon }$). We define the *sampling density* $q:= {s}/{n}$ and the *sampling radius* $\delta := {\varepsilon }/\sqrt{q}$. For a curve $\Gamma$, $l(\Gamma )$ denotes its length. For a subset of a surface $R\subset \mathcal{S}$, *a*(*R*) denotes the area of *R*. For sets $A, B\subset {\mathbb {E}}^3$, $A\oplus B$ denotes the Minkowski sum of *A* and *B*, i.e., the set $\{x+y:x\in A,\, y\in B\}$. For convenience, the special case $A\oplus B(0,r)$ shall be denoted by $A\oplus r$.

We next present some general lemmas, which will be needed in the proofs of the main lemmas.

**Level sets, boundary points and interior points** We now introduce some definitions which will play a central role in the analysis. First we define the notion of levels. Given facet $F\in \mathcal{S}$ and $k\ge 0$, define the *level set* $L_{\le k}:= F\cap (\partial F \oplus 2^k\delta )$, $L_{=k}:= L_{\le k}\setminus L_{\le k-1}$. For $x\in {{\mathcal {X}}}$, the *level* of *x*, denoted ${\text {Lev}}(x)$, is *k* such that $x\in L_{=k}$. Let $L_{\le k}({{\mathcal {X}}})$, $L_{=k}({{\mathcal {X}}})$ denote $L_{\le k}\cap {{\mathcal {X}}}$, $L_{=k}\cap {{\mathcal {X}}}$ respectively. Note that for $x\in L_{= k}$, $k\ge 1$, the distance $d(x,\partial F)\in (2^{k-1}\delta ,2^k\delta ]$. Hence, if ${\text {Lev}}(x)=k$, $D(x,2^{k-1}\delta )\subset F$. For $k=0$, $d(x,\partial F)\in [0,\delta ]$.

Next, we define a bi-partition of the point set into boundary and interior points. Given a point $x\in F$, *x* is a *boundary point* or $x\in {\text {Bd}}_F({{\mathcal {Y}}})$, if (i) $d(x,\partial F) \le \delta$, or (ii) there exists an empty disk (w.r.t. ${{\mathcal {Y}}}$) of radius greater than $2^{k-1}\delta$, whose boundary passes through *x*, where *k* is the minimum positive integer such that $d(x,\partial F) \in (2^{k-1}\delta ,2^k\delta ]$. The point *x* is an *interior point*, or $x\in {\text {Int}}_F({{\mathcal {Y}}})$, if and only if $x\in {{\mathcal {Y}}}\setminus {\text {Bd}}_F({{\mathcal {Y}}})$. In general, $x\in {\text {Bd}}_{\mathcal{S}}({{\mathcal {Y}}})$ if $x\in {\text {Bd}}_F({{\mathcal {Y}}})$ for some $F\in \mathcal{S}$; $x\in {\text {Int}}_{\mathcal{S}}({{\mathcal {Y}}})$ is defined similarly.

The above bi-partition induces a classification of potential Delaunay edges, depending on whether the end-points are boundary or interior points. Let $E_1$ denote the set of edges $\{x,y\}$ in ${\text {Del}}({{\mathcal {Y}}})$ such that $x,y\in {\text {Bd}}_{\mathcal{S}}({{\mathcal {Y}}})$. Let $E_2$ denote the set of edges $\{x,y\}$ in ${\text {Del}}({{\mathcal {Y}}})$ such that $x,y \in {\text {Int}}_{F}({{\mathcal {Y}}})$ for some $F\in \mathcal{S}$. Let $E_3$ denote the set of edges $\{x,y\}$ in ${\text {Del}}({{\mathcal {Y}}})$ such that $x,y \in {\text {Int}}_{\mathcal{S}}({{\mathcal {Y}}})$ and $x\in F$, $y\in F'$, $F,F'$ different facets in $\mathcal{S}$. Let $E_4$ denote the set of edges $\{x,y\}$ in ${\text {Del}}({{\mathcal {Y}}})$ with $x\in {\text {Bd}}_{\mathcal{S}}({{\mathcal {Y}}})$, $y\in {\text {Int}}_F({{\mathcal {Y}}})$, where *F* is a facet in $\mathcal{S}$.

Recall that the polyhedral surface $\mathcal{S}$ has *C* facets, with total area of its faces *A*, and total length of its boundaries *L*. We have the following lemmas, to be proven in Sect. 5.2.

### Lemma 5.1

$${\mathbb {E}}\,[\sharp (E_1)]\le O(1)\cdot \kappa ^2L^2s/A$$.

### Lemma 5.2

$${\mathbb {E}}\,[\sharp (E_2)] \le O(1)\cdot \kappa s$$.

### Lemma 5.3

$${\mathbb {E}}\,[\sharp (E_3)] \le O(1)\cdot (C-1) \cdot \kappa s$$.

### Lemma 5.4

$${\mathbb {E}}\,[\sharp (E_4)]\le O(1)\cdot \kappa ^2L^2s/A$$.

Given the above lemmas, the proof of Theorem 3.2 follows easily.

### Proof of Theorem 3.2

As in [4, Sect. 4], by Euler’s formula, the number of tetrahedra $t({\text {Del}}({{\mathcal {Y}}}))$ in the Delaunay triangulation of $\mathcal{S}$, is at most $e({\text {Del}}({{\mathcal {Y}}}))-\sharp ({{\mathcal {Y}}}) = e({\text {Del}}({{\mathcal {Y}}}))-s$, where $e({\text {Del}}({{\mathcal {Y}}}))$ is the number of edges in the Delaunay triangulation. Observe that the number of triangles in ${\text {Del}}({{\mathcal {Y}}})$ is also at most 4 times the number of tetrahedra. Therefore to bound the complexity of ${\text {Del}}({{\mathcal {Y}}})$ up to constant factors, it suffices to count the edges of ${\text {Del}}({{\mathcal {Y}}})$. Next, observe that any point $x\in {{\mathcal {Y}}}$ is either a boundary or an interior point, that is, ${\text {Bd}}_{\mathcal{S}}({{\mathcal {Y}}})\sqcup {\text {Int}}_{\mathcal{S}}({{\mathcal {Y}}})={{\mathcal {Y}}}$. An edge in ${\text {Del}}({{\mathcal {Y}}})$, therefore, can be either between two points in $\hbox {Bd}_{\mathcal{S}}({{\mathcal {Y}}})$, or two points in ${\text {Int}}_{\mathcal{S}}({{\mathcal {Y}}})$, or between a point in ${\text {Bd}}_{\mathcal{S}}({{\mathcal {Y}}})$ and another in ${\text {Int}}_{\mathcal{S}}({{\mathcal {Y}}})$. The case of a pair of points in ${\text {Int}}_{\mathcal{S}}({{\mathcal {Y}}})$ is further split based on whether the points belong to the same facet of $\mathcal{S}$ or different facets. Thus using the above exhaustive case analysis, the proof follows simply by summing the bounds. $\square$

Before proving Lemmas 5.1–5.4, we first present

### Some Technical Results

The following geometric and probabilistic lemmas prove certain properties of ${\varepsilon }$-nets on polyhedral surfaces and random subsets, as well as exploit the notion of boundary and interior points to get an exponential decay for boundary effects penetrating into the interior.

#### Proposition 5.5

[4] Let *F* be a facet of $\mathcal{S}$. For any Borel set $R \subset F$, we have

2 $$\begin{aligned} \frac{a(R)}{4\pi {\varepsilon }^2}&\le \sharp (R\cap {{\mathcal {X}}}) \le \frac{\kappa \,a(R\oplus {\varepsilon })}{\pi {\varepsilon }^2},\\&\mathrm{{ and~ therefore,}}\nonumber \end{aligned}$$

3 $$\begin{aligned} \frac{A}{4\pi {\varepsilon }^2}&\le \sharp (\mathcal{S}\cap {{\mathcal {X}}}) = n. \end{aligned}$$

#### Proposition 5.6

[4] Let *F* be a facet of $\mathcal{S}$, $\Gamma$ a curve contained in *F*, and $j\in {\mathbb {N}}$. Then

4 $$\begin{aligned} \sharp ((\Gamma \oplus j{\varepsilon })\cap {{\mathcal {X}}})&\le \frac{(2j+1)^2}{j}\,\kappa \,\frac{l(\Gamma )}{{\varepsilon }} \end{aligned}$$

5 $$\begin{aligned}&\le 9j\kappa \,\frac{l(\Gamma )}{{\varepsilon }} \quad \text { when } k\ge 1. \end{aligned}$$

The following fact can be easily verified using a greedy strategy.

#### Lemma 5.7

Given a circle $\Sigma _1\subset {\mathbb {E}}^2$ of unit radius centered at the origin, ***seven*** disks having centers in $\Sigma _1$ and radius 1/2, are necessary and sufficient to cover $\Sigma _1$.

**Fig. 1:**
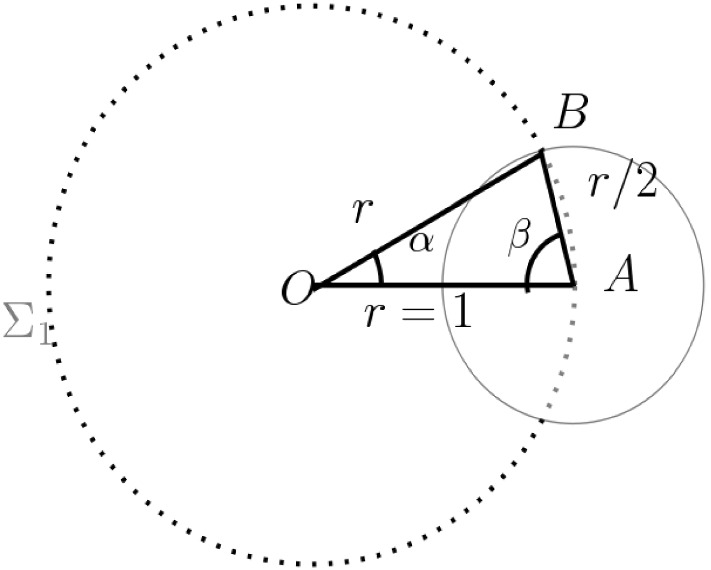
Angle covered by disk of radius 1/2 is $= 2\alpha$

The next lemma bounds the number of points of the original net ${{\mathcal {X}}}$, in a given level of $\mathcal{S}$ (defined with respect to the sampling radius $\delta$), in terms of the total length *L* of the facet boundaries, together with the sampling radius $\delta$ and some parameters related to the net ${{\mathcal {X}}}$.

#### Lemma 5.8

(level size)   $$\sharp (L_{= k}\cap {{\mathcal {X}}}) \le \sharp (L_{\le k}\cap {{\mathcal {X}}}) \le 9\kappa L\cdot {{2^k\delta }/{{\varepsilon }^2}}$$.

#### Proof

The first inequality is obvious, as $L_{=k}\subseteq L_{\le k}$. The proof of the second inequality follows by applying Proposition 5.6 over the boundaries of the facets. For a fixed facet $F\in \mathcal{S}$, we get

$$\begin{aligned} \sharp \,\biggl (\biggl (\partial F\oplus \frac{2^k\delta }{{\varepsilon }}\,{\varepsilon }\biggr ) \cap {{\mathcal {X}}}\biggr )\le \frac{9\kappa 2^k\delta }{{\varepsilon }}\cdot \frac{l(\partial F)}{{\varepsilon }}. \end{aligned}$$

Summing over all $F\in \mathcal{S}$, we get

$$\begin{aligned} \sharp (L_{\le k}\cap {{\mathcal {X}}})=\sum _{F\in \mathcal{S}}\sharp ((\partial F\oplus 2^k\delta )\cap {{\mathcal {X}}})\le \frac{9\kappa 2^k\delta }{{\varepsilon }^2}\sum _{F\in \mathcal{S}} l(\partial F)=\frac{9\kappa 2^k\delta }{{\varepsilon }^2}\,L. \end{aligned}$$

**Fig. 2:**
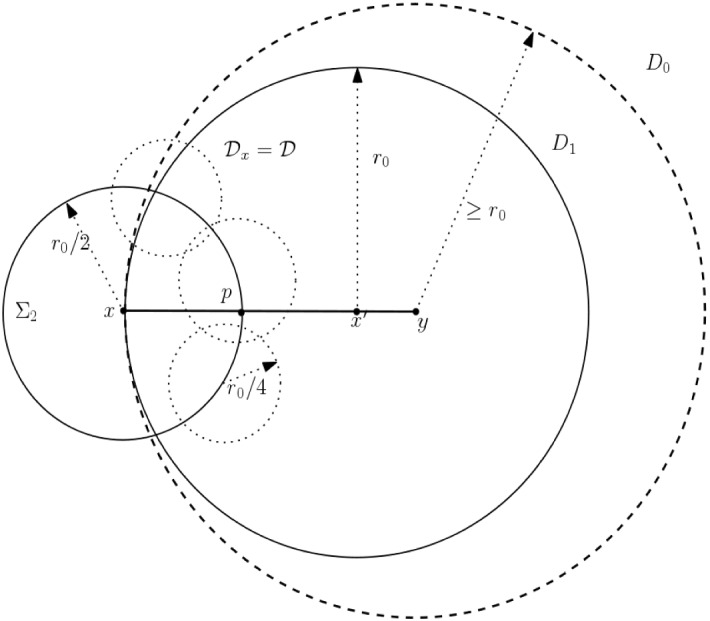
Illustration for Lemma 5.9

As mentioned previously, our goal is to bound the expected number of edges appearing in ${\text {Del}}({{\mathcal {Y}}})$ by bounding the probability that a given pair of vertices in ${{\mathcal {Y}}}$ appears as an edge. However, for a given pair of vertices there are infinitely many balls containing this pair on the boundary, and if any of these balls contains a point of ${{\mathcal {Y}}}$ in their interior, then the pair must form an edge in ${\text {Del}}({{\mathcal {Y}}})$. Bounding the above probability therefore seems to require us to forbid infinitely many events from occurring. The next couple of lemmas show how to restrict this to forbidding finitely many events, and obtain an exponentially decreasing bound on the probability of having long Delaunay edges.

Given a point *x* on some facet $F\in \mathcal{S}$ with supporting plane *P*, we call a disk $D \subset P$ a *supporting disk* for *x*, if the boundary $\partial D \ni x$ and ${{\mathcal {Y}}}\cap {{\,\mathrm{\mathrm{int}}\,}}D=\emptyset$, that is, the interior of the disk *D* does not contain any point of the random sample ${{\mathcal {Y}}}$. The following lemma shows that given any point *x* on a facet $F\in \mathcal{S}$ with supporting plane *P*, there exists a finite collection of sufficiently large discs in *F*, such that any large supporting disc for *x* must contain a disc from ${\mathcal {K}}_x$.

#### Lemma 5.9

Let *F* be a facet of $\mathcal{S}$ with supporting plane *P*, and $x\in F$ with ${\text {Lev}}(x)>0$. Then there exists a collection ${\mathcal {K}}_x$ of at most $c_B=7$ disks in *F* such that

(i) each $D\in {\mathcal {K}}_x$ is contained in *F*;
(ii) each $D\in {\mathcal {K}}_x$ has radius $r_0/4$, where $r_0 = 2^k\delta$ and $k\in {\mathbb {N}}$ is such that $0\le k<{\text {Lev}}(x)$; and
(iii) any supporting disk for *x* of radius at least $r_0$, contains at least one disk in ${\mathcal {K}}_x$.

#### Proof

Let $D_0\subset P$ be a supporting disk for *x*, with center $y\in P$ and radius $r\ge r_0$. Let $D_1=D(x',r_0)$ be the unique disk with center $x'$ on the line *xy*, radius $r_0$, and having $x\in \partial D_1$. Note that

- $D_1\subseteq D_0$ by construction, and
- $x'\in F$, since $r_0=2^{k}\delta \le 2^{{\text {Lev}}(x)-1}\delta$, so that $x'\in D(x,r_0)\subset F$.

Consider $\Sigma _2=\Sigma (x,r_0/2)$, and let $p=xx'\cap \Sigma _2$, that is, the point *p* lies on the line $xx'$, at distance $r_0/2$ from *x* (and therefore from $x'$ as well). We shall build a minimal covering ${\mathcal {K}}$ of the circle $\Sigma _2$, by disks centered in $\Sigma _2$, having radius $r_0/4$ (see Fig. 2 for the construction). From Lemma 5.7, we get $\sharp ({\mathcal {K}})= 7$. Let $D'\in {\mathcal {K}}$ be a disk in the covering. Then by the triangle inequality, $D'\subset D\,(x,r_0/2+r_0/4) \subset D(x,r_0)$. As before, by the definitions of ${\text {Lev}}(x)$ and $r_0$, this implies $D'\subset F$. Thus ${\mathcal {K}}$ satisfies conditions (i) and (ii) of the lemma. Further, since ${\mathcal {K}}$ is a covering of $\Sigma _2$, there exists $D_p\in {\mathcal {K}}$ such that $p\in D_p$. Therefore, the disk $D_p \subset D_1 \subset D_0$, and $D_p\subset F$. Thus $D_p\in {\mathcal {K}}$ satisfies condition (iii). Now taking ${\mathcal {K}}_x={\mathcal {K}}$ completes the proof of the lemma. $\square$

The following lemma gives a bound on the probability of having a large supporting disk, which falls exponentially as the radius of the disk increases. This will be crucial to our main proof, as it will be used in the proofs of all Lemmas 5.1–5.4. The idea is to use the construction of the previous lemma, together with the probabilistic bounds from Lemma 2.8.

#### Lemma 5.10

(Decay lemma) Given $x_1,\ldots , x_t \in {{\mathcal {X}}}$, such that ${\text {Lev}}(x_i)>0$, $1\le i\le t$, then for all $0\le k_i<{\text {Lev}}(x_i)$, with $r_i^*:=2^{k_i}\delta$, the probability of the event

$$\begin{aligned}&E\,:=\,\{\forall \,i\in [t],\;\exists \,D_i= D(y_i,r_i):\;\\&\quad \qquad \qquad \qquad \qquad \qquad r_i \ge r_i^*, \; x_i \in {{\mathcal {Y}}},\; D_i\text { is a supporting disk for } x_i\}, \end{aligned}$$

satisfies

$$\begin{aligned} {\mathbb {P}}[E]\le {\left\{ \begin{array}{ll} q^t &{}\text { if }k_{\max }=0,\\ c_1q^t\exp {({-}c_22^{2k_{\max }})} &{} \text { if }k_{\max }>0, \end{array}\right. } \end{aligned}$$

where $c_1=c_B^t$, $c_2 \ge 2^{-7}$, and $k_{\max } :=\max _i \{k_i\}$. Thus

$$\begin{aligned} {\mathbb {P}}[E]\le c_1q^t\exp {({-}c_22^{2k_{\max }})},\quad \ k_{\max }\ge 0. \end{aligned}$$

Note that we bound directly the joint probability of a collection of *t* vertices, simultaneously being chosen in ${{\mathcal {Y}}}$ and each of them having a supporting disk. This slight technical complication is required due to the fact that all these simultaneously occurring events are mutually dependent, as the random sample ${{\mathcal {Y}}}$ is chosen with a fixed size rather than choosing points independently. This is where the probabilistic Lemma 2.8 in handling such compound events proves to be so versatile.

#### Proof of Lemma 5.10

Firstly, consider the case where $k_{\max }=0$, i.e., all the $k_i$’s are zero. In this case we simply upper bound the probability of the event *E*, by the probability of including all the points $x_1,\ldots ,x_t$ in ${{\mathcal {Y}}}$. By Lemma 2.8, this is at most $q^t$.

We now come to the case when $k_{\max }>0$. Since for all $i\in [t]$, $k_i< \mathrm{Lev}(x_i)$, we can apply Lemma 5.9 for each *i*,with $k=k_i$, to conclude that for each *i*, there exists a collection ${\mathcal {K}}_{i}$ of at most $c_B$ disks of radius $r_i^*/4$, such that any supporting disk for $x_i$ having radius greater than $r_i^*$, must contain some disk $D_i^* \in {\mathcal {K}}_i$. Let *T* denote the set $\prod _{i=1}^t {\mathcal {K}}_i$. Let $B=(B_1,\ldots ,B_t)\in T$ denote a generic element of *T*. Taking the union bound over the set *T* gives the following:

$$\begin{aligned} {\mathbb {P}}[E]\,\le \, {\mathbb {P}}\,\bigl [\exists \, B \in T,\; \forall \, i \in [t]\,:\,B_i\cap {{\mathcal {Y}}}=\emptyset \bigr ]\,\le \,\sharp (T)\cdot {\mathbb {P}}\,[\forall \,i \in [t]:B_i\cap {{\mathcal {Y}}}=\emptyset ]. \end{aligned}$$

Let $j:= \arg \max _{i\in [t]}k_i$, so that $k_{\max }=k_j$. Now, the event *E* requires the set $x_1,\ldots x_t$ to be in the sample ${{\mathcal {Y}}}$, and the interiors of the disks $D_i^*$ to be free from points in ${{\mathcal {Y}}}$. In particular, the disk $D_j^*$ should not contain any points in ${{\mathcal {Y}}}$. Therefore applying Lemma 2.8 for the universe $C={{\mathcal {X}}}$, the random sample $A= {{\mathcal {Y}}}$, the included subset $B=\{x_1,\ldots ,x_t\}$, and the excluded subset $Z=D_j^*\cap {{\mathcal {X}}}$ of size at least $z=\pi (r_j^*/4)^2/({4\pi {\varepsilon }^2})$, we get

$$\begin{aligned} {\mathbb {P}}[E]&\,\le \,\sharp (T)\cdot {\mathbb {P}}\,[\forall \,B_i \in u:B_i\cap {{\mathcal {Y}}}=\emptyset ] \\&\,\le \, c_B^t q^t \exp {\biggl (\frac{-q}{2}\cdot \frac{\pi (r_j^*/4)^2}{4\pi {\varepsilon }^2}\biggr )}\le c_1q^t\exp {({-}2^{2k_{\max }-7})}, \end{aligned}$$

where in the last step we used the fact that $q = s/n$, $\delta = {\varepsilon }\sqrt{n/s}$, $r_j^*=2^{k_{\max }}\delta$, and set $c_1=c_B^t$. This completes the proof of the lemma. $\square$

The next lemma gives upper and lower bounds on the number of points in the original set ${{\mathcal {X}}}$, as well as the point sample ${{\mathcal {Y}}}$, contained in a disc in some facet of $\mathcal{S}$, as a function of the disc radius.

#### Lemma 5.11

(Growth lemma) Given any point $x\in \mathcal{S}$ in a facet *F*, and $0\le k<{\text {Lev}}(x)$, we have

- $$2^{2k-2}/q \le \sharp (D(x,2^k\delta )\cap {{\mathcal {X}}}) \le 4\cdot 2^{2k}/q$$,
- $$2^{2k-2} \le \sharp (D(x,2^k\delta )\cap {{\mathcal {Y}}}) \le 4\cdot 2^{2k}$$.

#### Proof

By the definition of ${\text {Lev}}(x)$, we have that $D(x,2^{{\text {Lev}}(x)-1}\delta )\subset F$. Now the statement follows by the application of Proposition 5.5:

$$\begin{aligned} \frac{\pi (2^{k}\delta )^2}{4\pi {\varepsilon }^2}&\le \sharp (D(x,2^k\delta )\cap {{\mathcal {X}}}) \le \frac{\pi (2^{k}\delta +{\varepsilon })^2}{\pi {\varepsilon }^2}, \end{aligned}$$

or

$$\begin{aligned} \frac{2^{2k-2}n}{s}&\le \sharp (D(x,2^k\delta )\cap {{\mathcal {X}}}) \le \frac{2\cdot 2^{2k}n}{s}, \end{aligned}$$

where $\delta := {\varepsilon }\sqrt{{n}/{s}}$. This gives the first statement of the lemma, with $q = s/n$. The second statement follows simply by taking expectation. $\square$

### Proofs of Lemmas 5.1–5.4

The proofs of Lemmas 5.1 and 5.2 now follow from the decay and growth lemmas, together with similar ideas as for the flat torus case.

#### Proof of Lemma 5.1

Let $x_1,x_2 \in {\text {Bd}}_{\mathcal{S}}({{\mathcal {Y}}})$. To bound the expected number of edges in $E_1$, we simply bound the number of pairs $(x_1,x_2)\in {\text {Bd}}_{\mathcal{S}}({{\mathcal {Y}}})\cdot {\text {Bd}}_{\mathcal{S}}({{\mathcal {Y}}})$. Let $l_1 :={\text {Lev}}(x)$ and $l_2:= {\text {Lev}}(y)$, and let $l:= \max _i (l_i)_{i=1}^2$. By definition, if $l = 0$, then $x_1,x_2\in {\text {Bd}}_{\mathcal{S}}({{\mathcal {Y}}})$. For $l \ge 1$, we get that $x_1\in {\text {Bd}}_{\mathcal{S}}({{\mathcal {Y}}})$ and $x_2\in {\text {Bd}}_{\mathcal{S}}({{\mathcal {Y}}})$ only if there exists a disk of radius at least $2^{l-1}\delta$ passing through $x_1$ or $x_2$, and containing no points of ${{\mathcal {Y}}}$. Therefore to bound the probability that $(x_1,x_2)\in ({\text {Bd}}_{\mathcal{S}}({{\mathcal {Y}}}))^2$, we can apply the decay Lemma 5.10, with $t=2$, for $i\in \{1,2\}$. We get

6 $$\begin{aligned} \begin{aligned} {\mathbb {P}}\,[(x_1,x_2)\in E_1]&\le {\mathbb {P}}\,\bigl [(x_1,x_2)\in ({\text {Bd}}_{\mathcal{S}}({{\mathcal {Y}}}))^2\bigr ]\\&\le c_1 q^2\exp {({-}c_2\cdot 2^{2l-2})} \le c_1 q^2\exp {({-}c_2'\cdot 2^{2l})}, \end{aligned} \end{aligned}$$

where $c_2'=c_2/4=2^{-9}$. Summing over all choices of levels of $x_1$ and $x_2$, we have

$$\begin{aligned} {\mathbb {E}}[\sharp (E_1)] \,\le \,\sum _{l_1\ge 0}\sharp (L_{=l_1}\cap {{\mathcal {X}}})\sum _{l_2\ge 0} \sharp (L_{=l_2}\cap {{\mathcal {X}}})\cdot {\mathbb {P}}\,\bigl [(x_1,x_2)\in ({\text {Bd}}_{\mathcal{S}}({{\mathcal {Y}}}))^2\bigr ]. \end{aligned}$$

By symmetry, it is enough to assume without loss of generality that $l_1\ge l_2$, i.e., $l=l_1$. Thus,

$$\begin{aligned} {\mathbb {E}}[\sharp (E_1)]\,\le \, 2\sum _{l_1\ge 0}\sharp (L_{=l_1}\cap {{\mathcal {X}}})\sum _{l_2= 0}^{l_1} \sharp (L_{=l_2}\cap {{\mathcal {X}}})\cdot {\mathbb {P}}\,\bigl [(x_1,x_2)\in ({\text {Bd}}_{\mathcal{S}}({{\mathcal {Y}}}))^2\bigr ]. \end{aligned}$$

Applying (6) and the level size Lemma 5.8, we get

$$\begin{aligned} {\mathbb {E}}[\sharp (E_1)]&\le 2\sum _{l_1\ge 0} \sharp (L_{\le l_1}\cap {{\mathcal {X}}}) \sum _{l_2=0}^{l_1} \sharp (L_{\le l_2}\cap {{\mathcal {X}}}) \cdot c_1q^2\exp {({-}c_2'\cdot 2^{2l_1})} \\&\le 2c_1q^2\sum _{l_1\ge 0} \frac{9\kappa L\cdot 2^{l_1}\delta }{{\varepsilon }^2}\sum _{l_2=0}^{l_1}\frac{9\kappa L\cdot 2^{l_2}\delta }{{\varepsilon }^2}\exp {({-}c_2'\cdot 2^{2l_1})} \\&\le 2c_1q^2\biggl (\frac{9\kappa L\delta }{{\varepsilon }^2}\biggr )^{2}\sum _{l_1\ge 0} 2^{l_1}\exp {({-}c_2'\cdot 2^{2l_1})}\sum _{l_2=0}^{l_1} 2^{l_2}. \end{aligned}$$

Using the definitions of *q* and $\delta$, together with Proposition 5.5, and writing the terms outside the summation as $N_1$, we obtain $N_1:= 2c_1q^2(9\kappa L\delta /{{\varepsilon }^2})^2=2c_1(9\kappa L)^2s/(n{\varepsilon }^2) \le 4c_1\cdot 4\pi (9\kappa L)^2s/{A}$ and continue

$$\begin{aligned} {\mathbb {E}}[\sharp (E_1)] \le N_1\sum _{l_1\ge 0} 2^{l_1}\exp {({-}c_2'2^{2l_1})}\cdot 2\cdot 2^{l_1} = 2N_1\sum _{l_1\ge 0} 2^{2l_1}\exp {({-}c_2' 2^{2l_1})}. \end{aligned}$$

The summation can be bounded using Lemma A.2 to get

$$\begin{aligned} {\mathbb {E}}[\sharp (E_1)]\le 2N_1\cdot 2\cdot \frac{\log \,(1/c_2')}{2ec_2'}=2N_1\cdot \frac{\log \,(1/c_2')}{e c_2'}. \end{aligned}$$

Now substituting $c_2'=2^{-9}$ gives ${\mathbb {E}}[\sharp (E_1)] \le 2\cdot 10^4c_1 s\cdot 4\pi (9\kappa L)^2/{A}$. $\square$

#### Proof of Lemma 5.2

Let *l* stand for $\min {\{{\text {Lev}}(x),{\text {Lev}}(y)\}}$. Observe that if $l=0$, then either *x* or *y* is a boundary point, and hence we can assume $l\ge 1$. Let $x'=\arg \min _{z\in \partial F} d(z,x)$, and $y'=\arg \min _{z\in \partial F} d(z,y)$, i.e., $x'$ is the closest point to *x* in $\partial F$, and similarly for $y'$. By the definition of ${\text {Bd}}_{\mathcal{S}}({{\mathcal {Y}}})$, observe that $d(x,y)\le d(x,x')+d(x',y)\le d(x,x')+d(y,y')\le 2\cdot 2^{l-1}\delta$. Hence we have that $d(x,y) \le 2^{l}\delta$.

By the growth Lemma 5.11, the expected number of Delaunay neighbors *y* of a point *x* such that $d(x,y)\le \delta$ is at most ${\mathbb {E}}[D(x,\delta )\cap {{\mathcal {Y}}}]\le q\cdot ({4}/{q})=4$. Thus the expected number of edges in $E_2$ from pairs (*x*, *y*) with $x,y\in {\text {Int}}_F({{\mathcal {Y}}})$ for some $F\in \mathcal{S}$, and $d(x,y)\le \delta$, is at most $4\cdot \sharp ({{\mathcal {Y}}})$. For longer-distance edges, let $k\ge 1$ be such that $2^{k-1}\delta \le d(x,y)\le 2^{k}\delta$. Taking $t=2$, $x_1=x$, $x_2=y$, $k_1=k-1$, and $k_2\le k_1$, and applying the decay Lemma 5.10, we get that

$$\begin{aligned} {\mathbb {P}}\,[\{x,y\}\in E_2]\le c_1q^2\exp \,(-c_2 2^{2k-2})\le c_1q^2\exp {({-}c_2'2^{2k})}, \end{aligned}$$

where $c_2'=c_2/4$. Summing over all possible choices of $l\ge 1$ and $k\le l$, we get

$$\begin{aligned} {\mathbb {E}}[\sharp (E_2)]&\,\le \,\sum _{l\ge 1} \sharp (L_{=l}\cap {{\mathcal {X}}}) \,\sum _{k=1}^{l} \sharp (D(x,2^{k}\delta )\cap {{\mathcal {X}}})\cdot c_1q^2\exp {({-}c_2' 2^{2k})}\\&\,\le \, c_1q^2\sum _{l\ge 1} \sharp (L_{=l}\cap {{\mathcal {X}}})\, \sum _{k=1}^{l} \kappa 2^{2k}(\delta /{\varepsilon })^2\exp {({-}c_2' 2^{2k})} \\&\,\le \, c_1\kappa q\,\frac{q\delta ^2}{{\varepsilon }^2}\sum _{l\ge 1} \sharp (L_{=l}\cap {{\mathcal {X}}})\cdot \frac{2\cdot (1/2)\cdot \log 1/c_2'}{ec_2'} \\&\,\le \, c_1\kappa qc_3\sum _{l\ge 1} \sharp (L_{=l}\cap {{\mathcal {X}}}) \,\le \, c_1\kappa qc_3 n \,=\, c_4\kappa s, \end{aligned}$$

where in the second step we applied the growth Lemma 5.11, and in the third step we bounded the sum $\sum _{k\ge 0} 2^{2k}\exp {({-}c_2 2^{2k})}$ using Lemma A.2 and the fact that $q = s/n = {\varepsilon }^2/\delta ^2$. Note that $c_3\le 4\cdot 10^3$, and $c_4 := c_1c_3\le 2\cdot 10^5$. $\square$

For the proofs of Lemmas 5.3 and 5.4, we need some more geometric ideas of [4].

#### Proof of Lemma 5.3

Let $x,y \in {\text {Int}}_{\mathcal{S}}({{\mathcal {Y}}})$, where $x\in F$ and $y\in F'$, for some $F,F' \in \mathcal{S}$. Let $F'$ be fixed. To analyze this case, we shall first give a geometric construction of [4], and state an observation from its proof.

**Fig. 3:**
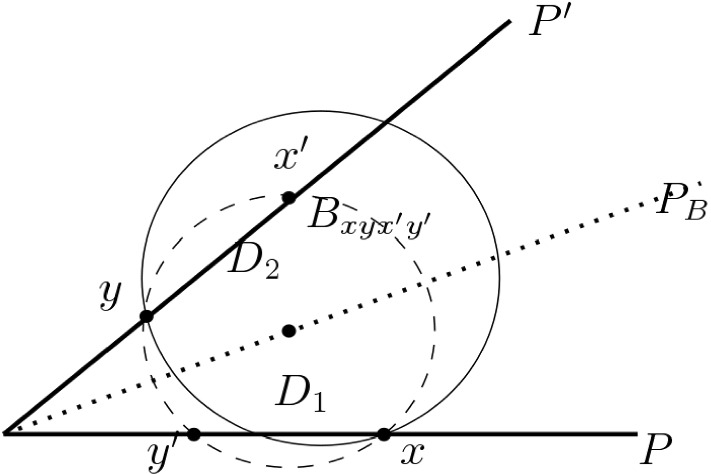
$x,y\in {\text {Int}}_{\mathcal{S}}({{\mathcal {Y}}})$, on different facets $F\subset P$, $F'\subset P'$

#### Construction 5.12

(Attali–Boissonnat [4])   Let *P* and $P'$ denote the supporting planes of the facets *F* and $F'$, respectively. Let $P_B$ be the bisector plane of *P* and $P'$. We denote by $x'\in P'$ the reflection of $x\in P$ with respect to $P_B$, and similarly, by $y'\in P$ the reflection of $y\in P'$. Let $B=B_{xyx'y'}$ be the smallest ball in ${\mathbb {E}}^3$ passing through $x,y,x',y'$, having intersections $D_1 = B\cap P$ and $D_2= B\cap P'$ with *P* and $P'$, respectively.

Attali and Boissonnat [4] observed that

#### Proposition 5.13

Any ball in ${\mathbb {E}}^3$ having *x* and *y* on its boundary, must contain either $D_1$ or $D_2$.

Therefore, if there exists a ball $B\in {\mathbb {E}}^3$ such that $x,y\in \partial B$, and ${{\mathcal {Y}}}\cap {{\,\mathrm{\mathrm{int}}\,}}B =\emptyset$, then either $D_1\cap {{\mathcal {Y}}}=\emptyset$ or $D_2\cap {{\mathcal {Y}}}=\emptyset$. We get

$$\begin{aligned} {\mathbb {P}}\,[\{x,y\}\in E_3]\le {\mathbb {P}}\,\bigl ([D_2\cap {{\mathcal {Y}}}= \emptyset ]\cup [D_2\cap {{\mathcal {Y}}}= \emptyset ]\bigr )\le 2\cdot {\mathbb {P}}\,[D_1\cap {{\mathcal {Y}}}= \emptyset ]. \end{aligned}$$

Observe that, as in Case II, we have $x'\in D(y,2^{{\text {Lev}}(y)}\delta )$, since otherwise $y\in {\text {Bd}}_{\mathcal{S}}({{\mathcal {Y}}})$. (Note that our definition of boundary points allows us to ignore the fact that $x'$ is not necessarily a point in ${{\mathcal {X}}}$.) Further, the set $\{x'\in D(y,2^{k}\delta )\}$, $0<k<{\text {Lev}}(y)$, is bounded in size by $\sharp (D(y',2^{k}\delta )\cap F \cap {{\mathcal {Y}}})$. The rest of the analysis for the fixed facet $F'$, therefore, follows as in Case II. Summing over all $F'\in \mathcal{S}\setminus \{F\}$, we get ${\mathbb {E}}[\sharp (E_3)]\le c_4(C-1)\kappa s$. $\square$

Before proving Lemma 5.4, we briefly describe a construction which will be central to our analysis.

#### Construction 5.14

(Attali–Boissonnat [4])   Let *P* be a plane and *Z* be a finite set of points. To each point $x\in Z$, assign the region $V(x)=V_x(Z)\subset P$ of points $y\in P$ such that the sphere tangent to *P* at *y* and passing through *x* encloses no point of *Z*. Let ${\mathcal {V}}:= \{V(x):x\in Z\}$.

We summarize some conclusions of Attali–Boissonnat regarding the construction. Proofs can be found in [4].

#### Proposition 5.15

(i) ${\mathcal {V}}$ is a partition of *P*.
(ii) For each $x\in Z$, *V*(*x*) is an intersection of regions that are either disks or complements of disks.
(iii) The total length of the boundary curves in ${\mathcal {V}}$ is equal to the total length of the convex boundaries.

#### Proof

The proofs of (i) and (ii) are easy.

We prove (iii). Consider a point $x\in Z$, and let *V*(*x*) be the region corresponding to *x* in ${\mathcal {V}}$. By (ii), $V(x) =\bigcap _{D \in D_x} D \cap \bigcap _{\bar{D}\in C_x} \bar{D}$, where $D_x$ is a set of disks and $C_x$ is a set of complements of disks in the plane *P*. Let $y\in \partial V(x)$. Then if $y\in \bigcap _{D \in D_x} D$, there exists $D_1\in D_x$ such that $y\in \partial D_1$, and so *y* is part of a convex segment in $\partial V(x)$. Otherwise, there exists $\bar{D_2}\in C_x$ such that $y\in \partial \bar{D_2}$. In this case, let *V*(*z*), $z\in Z$, denote the region such that $y\in \partial V(z)$. Then $D_2\supset V(z)$, and therefore *y* belongs to a convex segment in $\partial V(z)$. Thus, every point $y\in \partial V(x)$ is convex either for *V*(*x*) or for a neighboring region of *V*(*x*), and so the total length of the convex boundary curves in ${\mathcal {V}}$ gives the total length of all the boundary curves. $\square$

For the rest of this subsection, we shall apply Construction 5.14 for the plane *P* and the points in ${\text {Bd}}_{\mathcal{S}}({{\mathcal {Y}}})$ as *Z*. Let $\mathcal{T}:={\text {Int}}_{F}({{\mathcal {Y}}})$ for some facet $F\in \mathcal{S}$. Given $x\in Z$, $y\in P\setminus V(x)$, let $k_y=k_y(x)$ denote the least $k\ge 0$ such that $y\in \partial V(x)\oplus 2^k\delta$.

**Fig. 4:**
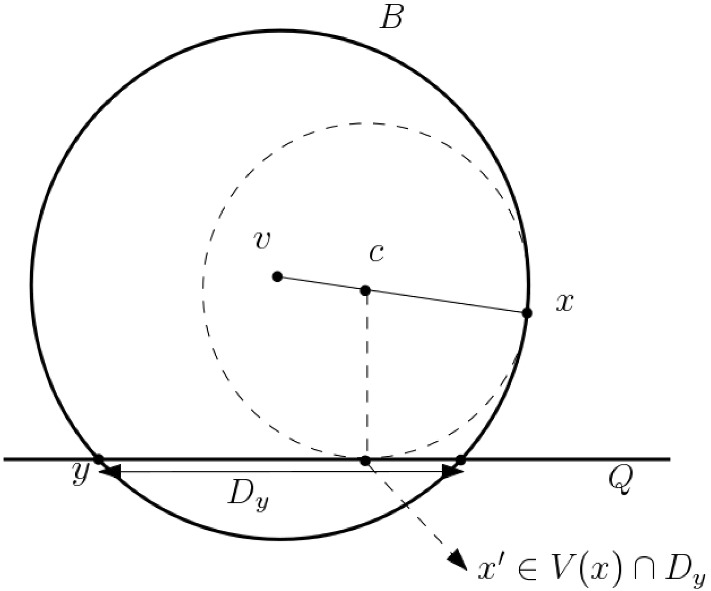
$$x\in Z={\text {Bd}}_{\mathcal{S}}({{\mathcal {Y}}})$$, $$y\in \mathcal{T}={\text {Int}}_{F}({{\mathcal {Y}}})$$, $$z\in V(x)\cap D_y$$

#### Proposition 5.16

(Attali–Boissonnat [4]) Suppose there exists a ball $B\subset {\mathbb {E}}^3$ and $y\in P$, such that $y,x\in \partial B$, and $B\cap \mathcal{T}= \emptyset$. Then the disk $D_y = P\cap B$ satisfies $D_y\cap \mathcal{T}=\emptyset$, $y\in \partial D_y$, and $D_y\cap V_x\ne \emptyset$.

#### Proof

The first part of the proposition, $D_y\cap \mathcal{T}=\emptyset$, follows from the condition on *B*. For the next part, note that $y\in \partial D_y$. Let *v* denote the center of the ball *B*, and let *z* be a variable point on the line segment *vx*. Let *B*(*z*) denote the ball with center *z*, having $x\in \partial B(z)$. For $z=v$, $B(z)=B$ intersects *P*. For $z=x$, $B(z)=\{x\}$ does not intersect *P*. Therefore there exists some value of $z=c$ such that *B*(*c*) is tangential to *P* (see Fig. 4). Let $x'$ denote the point where *B*(*c*) touches *P*. Then $x'\in D_y$, since by Construction 5.14, $B(z)\subset B$ for all *z* in the segment *vx*, and hence $B(c)\cap P \subset B\cap P$. Also, $x'\in V(x)$, by the definition of *V*(*x*). Therefore we get $x'\in D_y\cap V(x)$. $\square$

#### Lemma 5.17

If $\{x,y\}\in E_4$ with $x\in {\text {Bd}}_{\mathcal{S}}({{\mathcal {Y}}})$, $y\in {\text {Int}}(F)$, then $k_y\le {\text {Lev}}(y)$.

#### Proof

Suppose $\{x,y\}\in E_4$. Then there exists a ball $B\in {\mathbb {E}}^3$ with $x,y\in \partial B$, and ${{\mathcal {Y}}}\cap {{\,\mathrm{\mathrm{int}}\,}}B=\emptyset$. Therefore $D_y:= B\cap P$ also satisfies ${{\mathcal {Y}}}\cap {{\,\mathrm{\mathrm{int}}\,}}D_y=\emptyset$. By Proposition 5.16 we have that $D_y\cap V(x)\ne \emptyset$. Therefore, $y\in V(x)\oplus 2r_y$, where $r_y$ is the radius of $D_y$. But since $y\in {\text {Int}}(F)$, we have that any disk having *y* on its boundary and containing no point of ${{\mathcal {Y}}}$ in its interior can have radius at most $2^{{\text {Lev}}(y)-1}\delta$. Therefore $r_y\le 2^{{\text {Lev}}(y)-1}\delta$. Now taking $k_y$ such that $2^{k_y}\delta =2r_y$, we get $k_y \le {\text {Lev}}(y)$. $\square$

Now we partition the pairs of vertices $\{x,y\} \in E_4$ with $x\in {\text {Bd}}_{\mathcal{S}}({{\mathcal {Y}}})$, depending on whether $y\in V_F(x)$ or $y\in \partial V_F(x)\oplus 2^{k_y}\delta$. That is, given a facet $F\in \mathcal{S}$, let $E_4({\text {Int}}(F))$ denote the set of edges $\{x,y\}\in E_4$ with $y\in {{\,\mathrm{\mathrm{int}}\,}}V_F(x)$, and $E_4({\text {Bd}}(F))$ denote the set of edges in $E_4$ with $y\in \partial (V_F(x))\oplus 2^k\delta$, for $k\in [0,k_y]$. Define $E_4({\text {Int}}):= \bigcup _{F\in \mathcal{S}}E_4({\text {Int}}(F))$ and $E_4({\text {Bd}}) := \bigcup _{F\in \mathcal{S}} E_4({\text {Bd}}(F))$, respectively.

#### Proof of Lemma 5.4

The proof follows from Lemmas 5.18 and 5.19, which bound the expected number of edges in $E_4({\text {Int}})$ and $E_4({\text {Bd}})$ respectively. $\square$

#### Lemma 5.18

Given a facet $F\in \mathcal{S}$, ${\mathbb {E}}\,[E_4({\text {Int}}(F))]\le q\cdot \sharp ({{\mathcal {X}}}\cap F)$. As a consequence, ${\mathbb {E}}[E_4({\text {Int}})]\le s$.

#### Proof

Let $x\in {{\mathcal {X}}}$ and $y\in {{\mathcal {X}}}\cap F$. Let ${\mathcal {E}}_{x,y}$ denote the event $\{x,y\}\in E_4({\text {Int}}(F))$. Then ${\mathcal {E}}_{x,y}$ can occur only if (i) $x\in {\text {Bd}}_{\mathcal{S}}({{\mathcal {Y}}})$ and (ii) $y\in {\text {Int}}_{\mathcal{S}}(Y)\cap V_F(x)$. Fix a choice of ${{\mathcal {Y}}}$, say $Y\in {{{\mathcal {X}}}\atopwithdelims ()s}$. Conditioning on this choice of ${{\mathcal {Y}}}$, ${\text {Bd}}_{\mathcal{S}}({{\mathcal {Y}}})$ is a fixed set of points. The number of pairs contributing to $E_4({\text {Int}}(F))$ is at most $\sharp (\{(x,y)\in Y\times Y:x\in {\text {Bd}}_{\mathcal{S}}(Y),\,y\in V_F(x)\})$. The main observation is now that since ${\mathcal {V}}$ restricted to *F* is a sub-division of *F*, for each $y\in {{\mathcal {X}}}\cap F$, there is a unique $x=x_y\in {\text {Bd}}_{\mathcal{S}}(Y)$ such that $y\in V_F(x)$. Therefore we get

$$\begin{aligned} E_4({\text {Int}}(F))\,\le \sum _{V_F(x)\in {\mathcal {V}}\,:\,x\in {\text {Bd}}_{\mathcal{S}}(Y)}\sharp (V_F(x)\cap Y)\,\le \,\sharp (Y\cap F). \end{aligned}$$

Since the last bound holds for any choice of *Y*, taking expectation over all choices we get

$$\begin{aligned} {\mathbb {E}}\,[E_4({\text {Int}}(F))]\le {\mathbb {E}}\,[\sharp ({{\mathcal {Y}}}\cap F)]=q\cdot \sharp ({{\mathcal {X}}}\cap F). \end{aligned}$$

Now summing over all faces gives ${\mathbb {E}}[E_4({\text {Int}})]\le {\mathbb {E}}[\sharp ({{\mathcal {Y}}})] = s$. $\square$

#### Lemma 5.19

Given a facet $F\in \mathcal{S}$, ${\mathbb {E}}[E_4({\text {Bd}}(F))]\le O(1)\cdot \kappa ^2 Ls\cdot l(\partial F)/A$. As a consequence, ${\mathbb {E}}\,[E_4({\text {Bd}}(\mathcal{S}))]\le O(1)\cdot \kappa ^2 L^2s/A$.

#### Proof

To compute the expected value of $E_4({\text {Bd}}(\mathcal{S}))$, fix a face $F\in \mathcal{S}$. Consider a pair of points $x,y \in {{\mathcal {X}}}$ such that $y\in F$. Let ${\mathcal {E}}_{x,y}$ denote the event $\{x,y\}\in E_4({\text {Bd}}(F))$. The value of $E_4({\text {Bd}})$ is the number of $x,y\in {{\mathcal {X}}}$ such that ${\mathcal {E}}_{x,y}$ occurs. Taking expectations,

7 $$\begin{aligned} {\mathbb {E}}[E_4({\text {Bd}}(F))]\le \sum _{x\in {{\mathcal {X}}}}\sum _{y\in {{\mathcal {X}}}\cap F}{\mathbb {P}}[{\mathcal {E}}_{x,y}]. \end{aligned}$$

Observe that ${\mathcal {E}}_{x,y}$ occurs only if (i) $x\in {\text {Bd}}_{\mathcal{S}}({{\mathcal {Y}}})$ and (ii) $k_y(x)\le {\text {Lev}}(y)$, by applying Construction 5.14, for the plane *P*, $Z={\text {Bd}}_{\mathcal{S}}({{\mathcal {Y}}})$, $\mathcal{T}={{\mathcal {Y}}}\cap P$, and using Proposition 5.16. By Lemma 5.17, $k_y(x)\in [0,{\text {Lev}}(y)]$.

Let $P_{k_1,k_2}$ denote the probability that $\{x,y\}\in E_4({\text {Bd}}(F))$, with ${\text {Lev}}(x)=k_1$ and $k_y(x)=k_2$. Equation (7) can be rewritten in terms of $k_1$ and $k_2$ as

$$\begin{aligned} {\mathbb {E}}[E_4({\text {Bd}}(F))]\le \sum _{k_1\ge 0}\sharp (L_{=k_1}\cap {{\mathcal {X}}})\sum _{k_2\ge 0}\sum _{V_F\in {\mathcal {V}}}\sharp ((\partial V_F\oplus 2^{k_2}\delta ) \cap {{\mathcal {X}}}) \cdot P_{k_1,k_2}. \end{aligned}$$

Applying the decay Lemma 5.10 with $t=2$, $x_1=x$, $x_2=y$, $k_1=\max {\{0,k_1-1\}}$ (since $x\in {\text {Bd}}_{\mathcal{S}}({{\mathcal {Y}}})$), and $k_2=\max {\{0,k_2-1\}}$, we get

$$\begin{aligned} P_{k_1,k_2} \le c_1q^2\exp {({-}f(k^*))}, \end{aligned}$$

where $k^* := \max {\{0,k_1-1,k_2-1\}}$, and $f(k^*)= 0$ if $k^*=0$ and $c_2'2^{2k^*}$ otherwise, with $c_2'=c_2/4$.

As in the proof of Lemma 5.1, we shall use symmetry to handle the cases $k_1\ge k_2$ and $k_2 > k_1$ together. We get

$$\begin{aligned} {\mathbb {E}}\,[E_4({\text {Bd}}(F))]\le & {} 2\sum _{k_1\ge 0}\sharp (L_{=k_1}\cap {{\mathcal {X}}})\sum _{k_2\le k_1}\sum _{V(x)\in {\mathcal {V}}}\sharp ((\partial V(x)\oplus 2^{k_2}\delta ) \cap {{\mathcal {X}}})\\&\qquad \qquad \qquad \qquad \qquad \qquad \qquad \qquad ~~~~~~\times \,c_1q^2\exp {({-}c_2' 2^{2k_1})}. \end{aligned}$$

By the level size Lemma 5.8, we get that $\sharp (L_{=k_1}\cap {{\mathcal {X}}})\le {9\kappa L 2^{k_1}\delta }/{{\varepsilon }^2}$. Using Proposition 5.6, we get $\sharp (\{\partial V(x)\oplus 2^{k_2}\delta \}\cap {{\mathcal {Y}}}) \le {9\kappa \,2^{k_2}\delta \cdot l(\partial V(x))}/{{\varepsilon }^2}$. By Proposition 5.15 (iii), each boundary in the partition ${\mathcal {V}}$ is convex for some $x\in {\text {Bd}}_{\mathcal{S}}({{\mathcal {Y}}})$. Therefore we need to sum $l(\partial V(x))$ only over the convex curves in $\partial V(x)$, $x\in {\text {Bd}}_{\mathcal{S}}({{\mathcal {Y}}})$. The length of these curves is at most $l(\partial F)$. Thus we get

$$\begin{aligned} {\mathbb {E}}\,[E_4({\text {Bd}}(F))]\le 2L\cdot l(\partial F)\cdot \biggl (\frac{9\kappa \delta q}{{\varepsilon }^2}\biggr )^{2}\sum _{k_1\ge 0}2^{k_1}\sum _{k_2\le k_1}2^{k_2}c_1\exp {({-}c_2'2^{2k_1})}. \end{aligned}$$

Using Lemma A.2, the above summation is bounded by a constant. This results in $c_1\cdot O(1)\cdot {(9\kappa )^2L\delta ^2q^2\cdot l(\partial F)}/{{\varepsilon }^4} = O(\kappa ^2 Ls\cdot l(\partial F)/A)$, where the equality follows from the lower bound on *n* in (3) (Proposition 5.5), and the identities $q= s/n=\delta ^2/{\varepsilon }^2$. Summing *y* over all facets *F* in $\mathcal{S}$, we get ${\mathbb {E}}\,[E_4({\text {Bd}}(\mathcal{S}))]=O(1)\cdot {\kappa ^2 L^2s}/{A}$. $\square$

## Randomized Incremental Construction (Proof of Theorem 3.3)

In this section, we show how Theorems 3.1 and 3.2 imply bounds on the computational complexity of constructing Delaunay triangulations of ${\varepsilon }$-nets. Our main tool shall be Theorem 2.7. However, we need to show first that Condition 2.6 holds. The standard proof of this (see e.g. [10, 12], and also the discussion in [9, Sect. 2.2 D]) is sketched below.

Now we come to the proof of Theorem 3.3.

### Proof

To verify that Condition 2.6 indeed holds in the Euclidean metric case, observe first that the union $\mathcal{C}_p$ of the simplices in conflict with a new point *p* is a connected set. Therefore, walking on the adjacency graph of the simplices by rotating around the $(d-2)$-simplex shared between two adjacent faces on the boundary of $\mathcal{C}_p$, is enough to yield the set of new conflicts. This idea works directly when the Delaunay complex is embedded in the one-sheeted covering of ${\mathbb {T}}^d$. In the $3^d$-sheeted covering, there can be at most $3^{d^2}$ simplices formed using a given set of *d* points and *p*, and we need to check each of these possible simplices. Thus the time goes up by a multiplicative factor of $3^{d^2}$. However, as the increase is by a constant factor depending only on the dimension, Condition 2.6 is still satisfied, albeit with a larger constant. Now Theorem 2.7 can be applied to get the claimed result. $\square$

## Euclidean Orbifolds and Bounded-Distortion Metrics

In this section, we shall give some extensions of Theorems 3.1 and 3.3. The proofs of our theorems follow by finding covering spaces of bounded multiplicity where the Delaunay complex can be embedded, and generalizing Lemmas 4.5–4.8 to such spaces.

Given a space $\mathcal{S}$, ${\varepsilon }\in [0,1]$, and $\kappa \in {\mathbb {Z}}^+$, an $({\varepsilon },\kappa )$-*sample* is a set of points for which any ball of radius ${\varepsilon }$ in $\mathcal{S}$, contains at least one point and at most $\kappa$ points. The proof of Theorem 3.2 goes through for $({\varepsilon },\kappa )$-samples as well, using a generic $\kappa$ in place of the fixed value used in the proof. Similarly the proofs of Theorems 3.1 and 3.3 can also be translated to the $({\varepsilon },\kappa )$ setting. We have

### Theorem 7.1

Theorems 3.1, 3.2, and 3.3 hold when the point set ${{\mathcal {X}}}$ is an $({\varepsilon },\kappa )$-sample.

Our combinatorial results for uniformly random subsets of nicely-distributed point sets also hold when the subsets are chosen by independent sampling. The only change in the proof of Theorem 3.1 for independent sampling is when computing $P_p(k)$. We make use of the fact that points are selected independently, to get directly that $P_p(k) \le q^d (1-q)^{n_k} \le q^d\exp \,({-}qn_k)$. A similar adjustment is needed in the proof of Theorem 3.2. The rest of the proofs follow as before. Thus we get

### Theorem 7.2

Theorems 3.1 and 3.2 also hold in the case when the random sample is an i.i.d. sample with probability parameter $q=s/n$.

Coming to our results for Delaunay triangulations of Euclidean *d*-manifolds and embedded metrics in ${\mathbb {T}}^d$, we need a few definitions first.

**Euclidean** *d* **-orbifolds** A *d*-*dimensional Bieberbach group* $\mathcal{G}$ is a discrete group of isometries acting on ${\mathbb {E}}^d$. A *d*-*orbifold* ${\mathbb {E}}^d/\mathcal{G}$ is the compact quotient space (i.e., collection of orbits) of ${\mathbb {E}}^d$ acted on by a *d*-dimensional Bieberbach group $\mathcal{G}$. When the group action is free (i.e., has no fixed points), the *d*-orbifold is a closed Euclidean *d*-manifold. Every Euclidean *d*-manifold is the quotient space of some *d*-Bieberbach group acting on ${\mathbb {E}}^d$ [7, 32]. For Euclidean *d*-orbifolds we have:

### Theorem 7.3

Given a closed Euclidean *d*-orbifold ${\mathbb {M}}={\mathbb {E}}^d/\mathcal{G}$, equipped with the Euclidean metric, where $\mathcal{G}$ is a *d*-Bieberbach group, there exists a covering space $\mathcal{C}_{{\mathbb {M}}}$ with multiplicity $m = m^*(\mathcal{G},d)$, such that the Delaunay complex on ${\mathbb {M}}$ is a triangulation of $\mathcal{C}_{{\mathbb {M}}}$, and the statements of Theorems 3.1 and 3.3 apply for ${\varepsilon }$-nets, for any ${\varepsilon }\in [0,1/4]$.

### Proof

In this case, the existence of the covering space follows from the algorithmic version of Bieberbach’s theorem [6] by Caroli–Teillaud [11, Sect. 4].

### Theorem 7.4

Every Euclidean *d*-orbifold ${\mathbb {M}}$ has a covering space using a number $m_{{\mathbb {M}}}$ of sheets of a *d*-hyperparallelepiped $\tilde{{\mathbb {T}}}_{{\mathbb {M}}}^d$, where $m_{{\mathbb {M}}}$ depends only on *d*, such that the Delaunay triangulation of any point set on ${\mathbb {M}}$ is the projection of the Delaunay complex of the cover of the point set in the covering space.

The proof of Theorem 7.3 is along similar lines as that of Theorem 3.1, except (i) we work with the hyperparallelepiped $\tilde{{\mathbb {T}}}_{{\mathbb {M}}}^d$, and (ii) we need to take the effect of the multiplicity (i.e., the number $m_{{\mathbb {M}}}$ of sheets of $\tilde{{\mathbb {T}}}_{{\mathbb {M}}}^d$ required for Theorem 7.4 to hold) into account for all simplices. To handle (i), we observe that the volume of balls will change as the hyperparallelepiped is no longer a hypercube. Thus, a factor of the volume of the unit hyperparallelepiped $\tilde{{\mathbb {T}}}_{{\mathbb {M}}}^d$, will come into the estimates in Lemma 2.5. To handle the effect of multiplicity, we introduce an extra multiplicative factor of $m_{{\mathbb {M}}}^d$ in the bound of the number of possible *d*-simplices with any fixed set of points (compared to Lemma 4.8). Additionally, we take into account that the number of *distinct* points inside a potential Delaunay simplex is at least a $(1/m_{{\mathbb {M}}})$-fraction of the number guaranteed by Lemma 4.2. This gives a worse bound for the expected complexity of the star than in Theorem 3.1, but still a constant. $\square$

**Embedded metrics with bounded distortion** For a metric ${\mathfrak {d}}$ on some domain $\mathcal{S}$ embedded in ${\mathbb {E}}^d$, define its *distortion* $\kappa _{{\mathfrak {d}}}$ (with respect to ${\mathbb {E}}^d$) to be the minimum $\lambda \ge 1$ such that for all $x,y\in \mathcal{S}$,

$$\begin{aligned} \frac{\Vert x-y\Vert }{\lambda }\le {\mathfrak {d}}(x,y)\le \lambda \,\Vert x-y\Vert . \end{aligned}$$

A $d\times d$ matrix $M\in {\mathbb {E}}^d$ is *positive definite* if, for all $x\ne 0\in {\mathbb {E}}^d$, $x^\top Mx>0$. For a positive definite matrix *M*, define its *condition number* $c_M$ to be the ratio of its maximum to its minimum eigenvalue. For embedded metrics with bounded distortion, we have:

### Theorem 7.5

Given a metric ${\mathfrak {d}}$ over ${\mathbb {T}}^d$ with distortion $\kappa _{{\mathfrak {d}}}<\infty$, there exists an integer $m=m_{{\mathfrak {d}}}<(2\kappa _{{\mathfrak {d}}}\sqrt{d})^d$, such that the Delaunay triangulation over $({\mathbb {T}}^d,{\mathfrak {d}})$ embeds in ${\mathbb {T}}_m^d$ with the Euclidean metric. In particular, if ${\mathfrak {d}}$ is of the form ${\mathfrak {d}}(x,y) = \sqrt{(x-y)^\top M (x-y)}$, $x,y \in {\mathbb {T}}^d$, where $M\in {\mathbb {E}}^{d\times d}$ is a positive definite matrix having condition number at most $c_M$, then $m\le (2c_M\sqrt{d})^d$. Hence, given any ${\varepsilon }\in [0,1/4]$, the statements of Theorems 3.1 and 3.3 apply for ${\varepsilon }$-nets over the metric space $({\mathbb {T}}^d,{\mathfrak {d}})$.

For Theorem 7.5, we use a geometric condition of Caroli–Teillaud [11, Criterion 3.11] to explicitly bound the multiplicity of the covering space.

### Proof of Theorem 7.5

The action of ${\mathbb {Z}}^d$ on ${\mathbb {E}}^d$ is defined by translation, i.e., for $x\in {\mathbb {E}}^d$, $g\in {\mathbb {Z}}^d$, $gx := g\cdot x = g + x$. For a finite point set $P\in {\mathbb {E}}^d$, let $\Delta ({\mathbb {Z}}^d P)$ denote the largest ball in ${\mathbb {E}}^d$ containing no points from ${\mathbb {Z}}^d P$. Let $\delta ((k{\mathbb {Z}})^d)$ denote the minimum distance by which a point in ${\mathbb {E}}^d$ is translated by $(k{\mathbb {Z}})^d$. Finally, let $\pi (\,{\cdot }\,)$ denote the projection map of the covering space ${\mathbb {T}}_k^d$ onto ${\mathbb {T}}^d$. We shall use the following geometric condition of Caroli–Teillaud [11].

### Lemma 7.6

If $\Delta ({\mathbb {Z}}^d P) < \delta ((k{\mathbb {Z}})^d)/2$, then for any finite $Y\supset P$, the projection $\pi ({\text {Del}}({\mathbb {Z}}^d Y))$ is a triangulation of ${\mathbb {T}}^d$.

Now, observe that in the Euclidean metric, the diameter $\Delta _{\Vert \,{\cdot }\,\Vert }({\mathbb {Z}}^d P)$ of the largest ball not containing any point from the set ${\mathbb {Z}}^dP$ is at most $\sqrt{d}$, with equality holding when $\sharp (P)=1$, and that $\Delta (S') \le \Delta (S)$ for any $S'\supseteq S$, since adding points can only decrease the diameter of the largest empty ball. Therefore, in the metric ${\mathfrak {d}}$, we have that

$$\begin{aligned} \Delta _{{\mathfrak {d}}}({\mathbb {Z}}^dP) \le \Delta _{\Vert \,{\cdot }\,\Vert }({\mathbb {Z}}^dP)\cdot \max _{x,y\in \mathcal{S}}\frac{{\mathfrak {d}}(x,y)}{\Vert x-y\Vert }. \end{aligned}$$

By the previously observed bound on $\Delta _{\Vert \,{\cdot }\,\Vert }({\mathbb {Z}}^dP)$, we have

$$\begin{aligned} \Delta _{{\mathfrak {d}}}({\mathbb {Z}}^dP) \le \sqrt{d}\cdot \max _{x,y\in \mathcal{S}}\frac{{\mathfrak {d}}(x,y)}{\Vert x-y\Vert }. \end{aligned}$$

Also, letting $((k{\mathbb {Z}})^d)^*$ denote the non-identity elements of $(k{\mathbb {Z}})^d$,

$$\begin{aligned} \delta _{{\mathfrak {d}}}((k{\mathbb {Z}})^d) \,= \min _{x\in \mathcal{G},\; g\in ((k{\mathbb {Z}})^d)^*} {\mathfrak {d}}(x,gx) \,= \min _{x\in \mathcal{G},\; g\in ((k{\mathbb {Z}})^d)^*} \frac{{\mathfrak {d}}(x,gx)}{\Vert x-gx\Vert }\cdot \Vert x-gx\Vert . \end{aligned}$$

For the flat torus ${\mathbb {T}}_k^d$, $\min _{x,g} \Vert x-gx\Vert = k$. Therefore, in the metric ${\mathfrak {d}}$, the condition of Lemma 7.6 is satisfied if

$$\begin{aligned} \sqrt{d} \cdot \max _{x,y\in {\mathbb {Z}}^d P} \frac{{\mathfrak {d}}(x,y)}{\Vert x-y\Vert }<\,\frac{1}{2}\cdot \min _{x\in P,\,g\in ((k{\mathbb {Z}})^d)^*} \frac{{\mathfrak {d}}(x,gx)}{\Vert x-gx\Vert }\cdot \Vert x-gx\Vert . \end{aligned}$$

This gives

$$\begin{aligned}&2\sqrt{d}\cdot \max _{x,y\in {\mathbb {Z}}^d P} \frac{{\mathfrak {d}}(x,y)}{\Vert x-y\Vert }\cdot \max _{x'\in P,\, g\in ((k{\mathbb {Z}})^d)^*} \frac{\Vert x'-gx'\Vert }{{\mathfrak {d}}(x',gx')}<k, \end{aligned}$$

which is true if

$$\begin{aligned}&2\sqrt{d}\cdot \max _{x,y\in {\mathbb {Z}}^d P} \frac{{\mathfrak {d}}(x,y)}{\Vert x-y\Vert }\cdot \min _{x',y'\in {\mathbb {Z}}^d P} \frac{{\mathfrak {d}}(x',y')}{\Vert x'-y'\Vert } <k,\\ \end{aligned}$$

or

$$\begin{aligned}&2\sqrt{d}\cdot \kappa _{{\mathfrak {d}}} < k, \end{aligned}$$

since by definition, the distortion $\kappa _{{\mathfrak {d}}}$ satisfies for all $x,y\in {\mathbb {Z}}^d P$, ${\mathfrak {d}}(x,y)\le \kappa _{{\mathfrak {d}}}\Vert x-y\Vert$, i.e., $\kappa _{{\mathfrak {d}}} \ge \max _{x,y\in {\mathbb {Z}}^d P}{{\mathfrak {d}}(x,y)}/{\Vert x-y\Vert }$, as well as $\Vert x-y\Vert \le \kappa _{{\mathfrak {d}}}{\mathfrak {d}}(x,y)$, i.e., $\kappa _{{\mathfrak {d}}}\ge \min _{x,y\in {\mathbb {Z}}^d P} {{\mathfrak {d}}(x,y)}/{\Vert x-y\Vert }$.

Since the fundamental domain of ${\mathbb {T}}_k^d$ contains $k^d$ copies of the fundamental domain of ${\mathbb {T}}^d$, we have $m \le k^d$, and so the first part of the theorem follows with $m\le (2\sqrt{d}\kappa _{{\mathfrak {d}}})^d$. The second part easily follows from the Courant minimax principle, i.e., that (a) $\max _{\Vert x\Vert =1} \{x^\top M x\}=\max _{\Vert x\Vert =1}\{ \Vert Ax\Vert \} = \sigma _{\max }(A)$, and that (b) $\min _{\Vert x\Vert =1} \{x^\top Mx\} = \sigma _{\max }(A^{-1}) =\sigma _{\min }(A)$ where *A* is such that $M=A^\top A$ and $\sigma _{\max }(A)$, $\sigma _{\min }(A)$ are respectively the largest and the smallest singular values of *A*. $\square$

## Conclusion and Remarks

In this paper, we analyzed the behavior of the usual RIC algorithm for the Delaunay triangulation of nice point sets, focusing on the cases where the ambient space is the flat *d*-torus or a polyhedral surface in ${\mathbb {E}}^3$. Similar questions can be asked for other spaces where the Delaunay triangulation is known to have low complexity for “nice” point sets.

We leave for further research a more general analysis of RIC of Delaunay triangulations of cases such as polyhedral surfaces in higher dimensions, as well as extending the techniques developed in this paper to the RIC of other geometric problems.

## Appendix

### Proofs from Section 1

#### Lemma A.1

Given ${\varepsilon }\in (0,1]$, an ${\varepsilon }$-net ${{\mathcal {X}}}$ over ${\mathbb {T}}^d$, of *n* points, a uniformly random sample $S\subset {{\mathcal {X}}}$ of *k* points and ${\varepsilon }'={\varepsilon }\root d \of {n/k}$, then:

(i) With high probability, there exists a ball of radius ${\varepsilon }'$ with $\Omega (\log k/\log \log k)$ points in *S*.
(ii) With probability at least a constant, there exists a ball of radius $\Omega ({\varepsilon }'\log k)$ with no points in *S*.

#### Proof (sketch)

The following balls-and-bins and coupon-collector arguments can be shown to prove what follows:

(i) Assuming the ${\varepsilon }$-net is over the unit cube or unit ball in ${\mathbb {E}}^d$, a volume argument gives that there are $\Omega ({\varepsilon }'^{-d}) = \Omega (k)$ disjoint balls of radius ${\varepsilon }'$, which will be our “bins”. Now choosing *k* points from the *n* points in the ${\varepsilon }$-net is akin to throwing *k* packets into *k* bins—with high probability, the maximum load of a bin is $\Omega (\log k/\log \log k)$, i.e., there exists a ball of radius ${\varepsilon }'$ with $\Omega (\log k/\log \log k)$ points.
(ii) Similarly, there are $\Omega ({\varepsilon }'/\log k)=\Omega (k/\log k)$ balls of radius ${\varepsilon }'\root d \of {\log k}$ that cover the ${\varepsilon }$-net, and choosing *k* points from this covering can be thought of as an instance of the coupon collector problem: we are allowed *k* draws (i.e., we choose *k* points), each draw is one of the “coupons” (i.e., the balls in the covering) with equal probability, and we want the probability that at least one coupon has not been collected, i.e., at least one ball from the covering has no point chosen from it. Now the well-known lower tail of the number of draws taken to collect all coupons in the coupon collector problem, gives that with high probability, for $k'=\Omega (k/\log k)$ coupons, and at most $O(k'\log k') = k$ draws, all coupons will not be collected, that is, some ball of radius $\Omega ({\varepsilon }'\root d \of {\log k})$ will be empty. $\square$

### Proofs from Section 5

#### Lemma A.2

Given $a>0$, $b\in (0,1)$, the sum

$$\begin{aligned} \sum _{n\in {\mathbb {Z}}_+} 2^{an}\exp \,({-}b2^{an})\le \frac{2\log _2 (1/b)}{eab}. \end{aligned}$$

#### Proof

The proof follows from elementary calculus. The maximum term is when $2^{an}=1/b$, i.e., $n={\log _2(1/b)}/{a}$, and evaluates to $2^{an}\exp \,({-}b2^{an})={1}/{eb}$, and terms decrease exponentially afterwards. The sum is therefore upper bounded by twice the maximum term, times the number of terms before the maximum, which is the claimed bound. $\square$
